# Data‐Driven Cation Engineering Guides Electrolyte Design for Sustainable Aqueous Zinc Battery Chemistries

**DOI:** 10.1002/adma.202522059

**Published:** 2026-04-08

**Authors:** Xuesong Xie, Yinfei Lyu, Huorong Ren, Witold Pedrycz, Yifan Li, Yang Yang, Xuehai Tan, Minggang Xie, Yi Guan, Yuxuan Xue, Ning Chen, Zhi Li

**Affiliations:** ^1^ Department of Chemical and Materials Engineering University of Alberta Edmonton Alberta Canada; ^2^ School of Electro‐mechanical Engineering Xidian University Xi'an China; ^3^ Department of Electrical and Computer Engineering University of Alberta Edmonton Alberta Canada; ^4^ Hard X‐Ray Micro Analysis BL Canadian Light Source Saskatoon Saskatchewan Canada

**Keywords:** aqueous zinc‐ion batteries, cation‐engineered electrolytes, data‐driven framework, failure mechanism, vanadium dissolution

## Abstract

Vanadium oxides have emerged as attractive cathode materials for zinc‐based batteries owing to their high theoretical capacity and versatile redox chemistry. Nevertheless, their persistent dissolution in aqueous electrolytes remains a long‐standing challenge, hindering real‐world implementation. Here, we develop a cation‐engineered electrolyte strategy enabled by a data‐driven framework that integrates density functional theory (DFT) calculations, discrete wavelet transform (DWT)‐based multi‐scale analysis, and differential feature extraction, to efficiently screen potential hetero‐cations and their combinations with objective statistic quantification, while minimizing trial‐and‐error experimentation and selection bias. As a proof of concept, the Zn/VO*x* batteries with the predicted Na^+^‐Mg^2+^‐Zn^2+^ tri‐cation electrolyte (NMZ) achieved exceptional reversibility and record‐long cycling stability, sustaining 500 cycles at 0.2 A g^−1^ (1400 h) and 10,000 cycles at 5 A g^−1^. The tri‐cation electrolyte successfully triggers a potential‐driven sequential ion insertion pathway involving Na^+^, Mg^2+^, and Zn^2+^, thereby fundamentally suppressing proton intercalation above 1.3 V and hydrated Zn^2+^ insertion near 1.0 V (vs Zn^2+^/Zn). This work not only provides valuable data‐driven insights into ion‐engineering electrochemistry for regulating insertion stability but also uncovers critical ion‐related factors that are frequently overlooked. This approach establishes a reusable and statistically robust framework for guiding research across diverse battery chemistries.

## Graphical abstract

To address the persistent challenges of vanadium dissolution and capacity fading in zinc‐ion batteries, we introduce a cation‐engineered electrolyte strategy underpinned by a data‐driven framework. This approach efficiently and reliably predicts a Na^+^‐Mg^2+^‐Zn^2+^ tri‐cation electrolyte with well‐balanced and sustainable advantages, enabling Zn/VO*_x_* batteries to achieve exceptional electrochemical reversibility and record‐long stability.

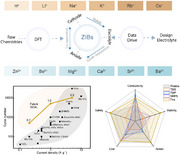

## 1 Introduction

Environmental sustainability and resource availability have become increasingly important considerations for next‐generation energy storage, propelled by the exponential growth of smart grids and electric vehicle adoption. Zinc ion batteries (ZIBs), noted for their substantial cost‐competitiveness and compatible redox properties, stand out compared with Li‐, Na‐, K‐, Ca‐, Mg‐, and Al‐based aqueous batteries. However, their large‐scale development remains hindered by limited cycle life and elusive failure mechanism [1, 2]. As one of the most widely adopted cathodes for ZIBs, vanadium oxides achieved practically relevant benchmarks (above 15 mg cm^−2^ mass loading and up to 10 A g^−1^ rates) [3, 4], with abundant resources and oxidation states [5]. Nevertheless, their persistent dissolution in aqueous electrolytes—particularly under low‐current density conditions (≤ 1.0 A g^−1^, Table S1)—casts doubt on the practical large‐scale development of this technology, despite the ostensibly superior cycling stability reported at current densities exceeding 10 C [5, 6, 7].

To date, no mechanism‐driven strategies are available to sustain capacity stability in vanadium oxide‐based ZIBs while fully harness the inherent advantages of aqueous media. Some promising experimental studies have demonstrated that pre‐insertion of cations (Li^+^ [8], Na^+^ [9], NH_4_^+^ [10], Ca^2+^ [11], Zn^2+^[5, 6, 7], Al^3+^, etc.), H_2_O, and polymer molecules [12, 13, 14], into bulk cathodes can facilitate ion diffusion and reinforce structural stability through electrochemical shielding and physical support [15]. However, pre‐inserted cations inevitably reduce the initial oxidation state of vanadium and occupy available intercalation sites. Despite some improvements, the capacity fading of vanadium oxides persists when tested under low‐current‐density operation [7, 16]. Meanwhile, non‐electrode‐intrinsic approaches and passive blocking strategies, including electrolyte additives and surface coatings, face inherent limitations, particularly under high‐rate conditions. We identify the key challenge as the absence of rational design principles grounded in a mechanistic understanding of ion‐engineering electrochemistry, necessitating systematic theoretical and experimental studies to elucidate the underlying structure‐property‐performance relationships. Rapid advances in computer science have rendered data‐driven frameworks, including machine learning, statistical modeling, and multivariate analysis, increasingly accessible for extracting meaningful patterns and guiding rapid, predictable research trajectories. These tools are highly effective in enabling the rational design of electrolytes, anodes, and cathodes across multicomponent systems.

**FIGURE 1:**
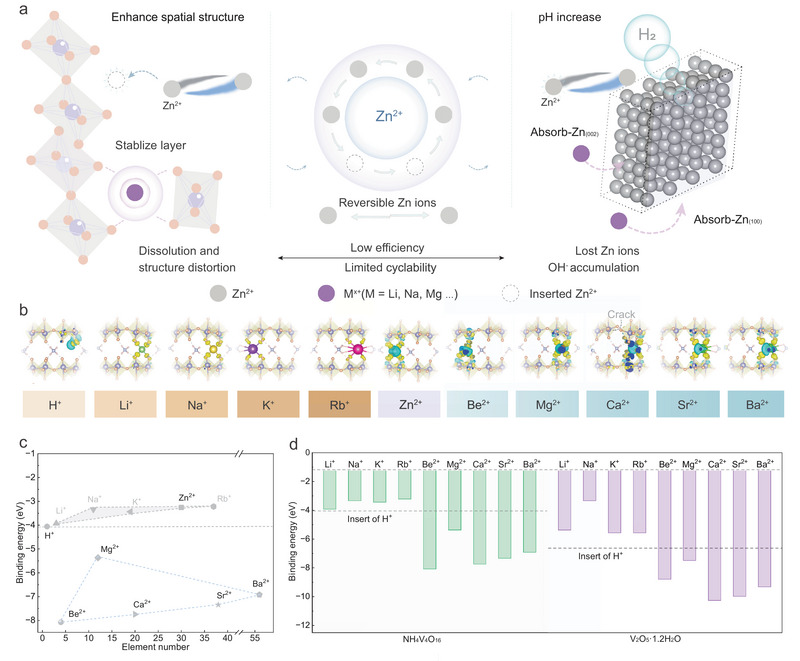
Schematic illustration and theoretical simulations. (a) Nanoscale spatial insertion states, energy storage mechanism, and dissolution behavior in layered vanadium oxide‐based ZIBs. (b) Differential charge density distributions of various cations in the NVO cathode. (c) Correlation between atomic number and binding energy in NVO. (d) Comparison of binding energy levels in the NVO and VO cathodes.

Herein, we present a data‐driven, multi‐scale methodology that integrates DFT calculations with DWT‐based multiscale analysis of cyclic voltammetric (i‐E) data to enable trend identification and fine‐grained feature extraction. This framework facilitates quantitative, statistically robust assessment of hetero‐cations through objective metrics (median, inter‐quartile range, outlier frequency) and sensitively predicts their applicability for advancing sustainable vanadium oxide‐based ZIB chemistries. Spatial modeling and energy binding analysis revealed that a regular triangular relationship emerged between the VO*_x_* host and both alkali and alkaline earth metal cations. As a proof of concept, the predicted tri‐cation Na^+^‐Mg^2+^‐Zn^2+^ (NMZ) electrolyte, identified from single‐ion features, enables Zn/VO*_x_* batteries to achieve exceptional electrochemical reversibility and record‐breaking cycling stability (Figure 1a), completing prospective validation of this framework. Additionally, the tri‐cation NMZ electrolyte triggers a sequential gradient ion‐insertion process that fundamentally suppresses deteriorated proton insertion at near or above 1.3 V and hydrated Zn^2+^ insertion around 1.0 V through the replacement insertion of Na^+^ at around 1.2 V and single Mg^2+^ intercalation at near 1.07 V (vs. Zn^2+^/Zn). This study establishes a robust foundation for uncovering the mechanisms of ion‐engineering electrochemistry through a data‐driven, multi‐scale approach that integrates automated feature extraction with quantified decision‐making reliability.

## 2 Results

A comprehensive analysis of cations, including H^+^, Li^+^, Na^+^, K^+^, Rb^+^, Be^2+^, Mg^2^^+^, Ca^2^^+^, Sr^2+^, and Ba^2+^ ions, was conducted using DFT calculations to systematically investigate their electron‐transfer characteristics, binding energies, and spatial ion‐intercalation configurations (Figure 1b). The pronounced electron localization and high binding energy (−4.07 eV) accompanying proton insertion promote selective cleavage of the O‐V coordination bonds (Figure 1c). The alkali metal cations Li^+^ and Na^+^ are uniformly coordinated within the hexagonal interlayer architecture in the NH_4_V_4_O_10_ (NVO) cathode, which may be attributed to their favorable ionic radii (0.76 Å for Li^+^, and 1.02 Å for Na^+^) [17]. In contrast, the larger K (1.38 Å) and Rb^+^ (1.52 Å) ions exhibit additional interactions with NH_4_^+^ molecules. This phenomenon originates from the diffuse nature of their outer‐shell orbitals [18], which facilitates electron delocalization and introduces steric hindrance, thereby obstructing sequential ion insertion into interlayer sites.

For Zn^2+^ (0.74 Å), the uniform charge density distribution suggests superior compatibility compared with other dual‐cations. This finding implies favorable theoretical structural stability, challenging the conventional view that the pronounced polarization of Zn^2+^ tends to disrupt V‐O bonds [19]. Be^2+^ exhibits electron density delocalization within a three‐dimensional coordination environment toward neighboring O‐V bonds and possesses the most negative binding energy of −8.08 eV (Figure 1c). Mg^2+^ insertion results in a tetra‐coordinated configuration, similar to those of Ca^2+^, Sr^2+^, and Ba^2+^, but it displays the least negative binding energy (−5.37 eV) among these ions. Ca^2+^ exhibits a binding energy of −7.74 eV and induces a noticeable structural break between adjacent corner‐sharing VO_6_ octahedra, suggesting unfavorable spatial compatibility upon insertion. The incorporation of both Sr^2+^ and Ba^2+^ induces additional electron density accumulation around the NH_4_^+^ species located within the interlayer space. For both alkali and alkaline‐earth metal ions, the binding energies exhibit a characteristic triangular dependence on cation atomic number, reflecting their relative thermodynamic stability and propensity for spatial embedding. Meanwhile, the binding energies in the VO (V_2_O_5_·1.2H_2_O) cathode follow a similar regulatory trend (Figure 1d and Figure S1).

Guided by theoretical insights described above, we developed a data‐driven, multi‐scale analytical framework to systematically elucidate the influence of hetero‐cations under electrochemical driving conditions. Cyclic voltammetry (CV) curves of a battery typically exhibit hierarchical electrochemical information, including trends, perturbations, and localized nonlinear features. However, conventional methods are ill‐suited to concurrently capture these features. Accordingly, the current‐potential (*i*‐*E*) trajectory was decomposed into frequency‐ and resolution‐specific components using discrete wavelet transform (DWT)‐based multiscale analysis. This approach harnesses the inherent multi‐resolution decomposition capability to simultaneously resolve macroscale trends and microscale transient features across multiple scales. Let *x*(*t*) denote a cyclic voltammetry (*i*‐*E*) trajectory containing *N* complete electrochemical cycles. The sampling time window of the *i*‐th cycle (*i* = 1, …, *N*) is denoted by $W_{i}=\left[t_{i}^{start},t_{i}^{end}\right]$.

The original *i‐E* curve is decomposed via DWT into approximation and detail components across multiple scales, representing the global trend and local fluctuations, respectively. Inspecting the overall shapes of these component curves provides a preliminary insight into stability and abnormal fluctuations. At the terminal scale *J*, *x*(*t*) it can be decomposed as follows:

(1) $$x\left(t\right)=A_{J}\left(t\right)+\sum_{j=1}^{J}D_{j}\left(t\right)$$

where the approximation and detail components are reconstructed from the wavelet coefficients as follows:

(2) $$A_{J}\left(t\right)=\sum_{k}a_{J,k}\;\phi_{J,k}\left(t\right),\;D_{j}\left(t\right)=\sum_{k}d_{j,k}\;\psi_{j,k}\left(t\right)$$

Here,*a*_*J*,*k*_ and*d*_*j*,*k*_ denote the approximation and detail coefficients at each scale, whereas ϕ_*J*,*k*_(*t*) and ψ_*j*,*k*_(*t*) represent the corresponding wavelet basis functions. The number of decomposition levels *J* depends on both the signal length and the selected decomposition scale, and directly determines the total number of coefficients obtained.

For convenience, the reconstructed components are denoted as follows:

(3) $$X_{A,j}\left(t\right)=A_{j}\left(t\right),\;X_{D,j}\left(t\right)=D_{j}\left(t\right)$$

where τ ∈ *A*, *D* indicates the component type (approximation/detail). Accordingly, the reconstruction of each decomposed component directly relies on both the quantity and completeness of the wavelet coefficients obtained at each level.

**FIGURE 2:**
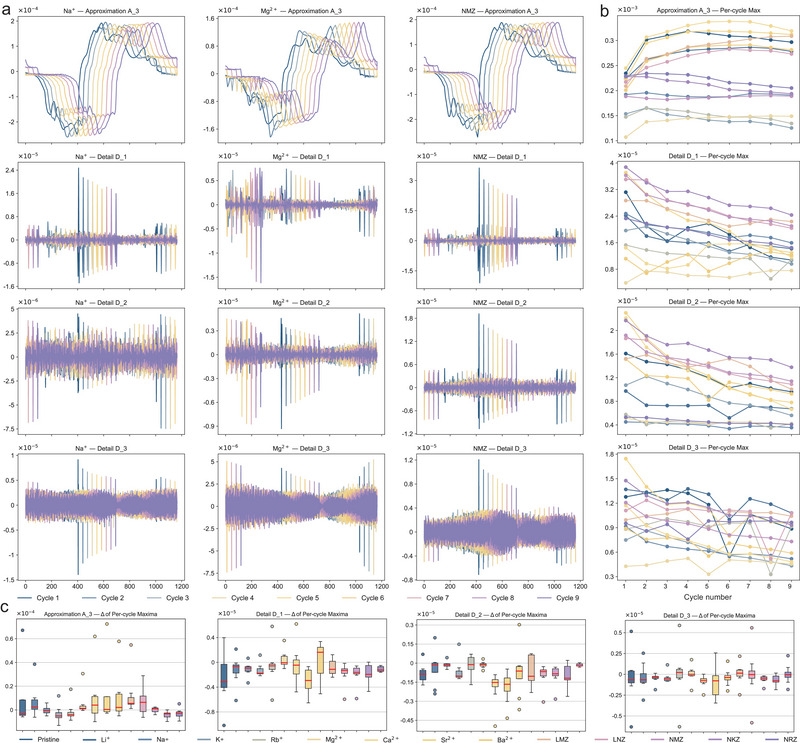
Multiscale decomposition and analysis. (a) Multiscale decomposition of CV curves for Na^+^‐, Mg^2+^‐, and NMZ‐based electrolytes, respectively. (b) Multiscale evolution of maxima and stability across successive cycles for different cation electrolytes. (c) Box‐plot distributions of maxima and stability analysis of the electrolyte systems.

The approximation and detail components across multiple scales were extracted to reflect stability and reversibility characteristics (Figure 2a and Figure S2). In the low‐frequency components (approximation A_3), electrolytes containing Li^+^, Na^+^, and Mg^2+^ show highly overlapping inter‐cycle curves with negligible fluctuations, indicating robust trend consistency and long‐term stability—key indicators of superior stability. In contrast, the pristine Zn^2+^ and other cation‐containing electrolytes exhibit pronounced spectral shifts. The detail components (D_1‐3) of the pristine, Rb^+^, Ca^2+^, Sr^2+^, and Ba^2+^ reveal frequent spikes and irregular oscillations, indicative of the microstructural instability and progressive electrochemical degradation. Based on the above analysis, cation combinations were further employed to predict their specific compatibility. LMZ (Li^+^‐Mg^2+^‐Zn^2+^) and LNZ (Li^+^‐Na^+^‐Zn^2+^) exhibit relatively ordered variations in the detail components, accompanied by a reduction in the low‐frequency A_3 components. By contrast, NKZ (Na^+^‐K^+^‐Zn^2+^) and NRZ (Na^+^‐Rb^+^‐Zn^2+^) exhibit the opposite variation pattern. Across both the high‐frequency detail components (D_1‐3) and the low‐frequency components, the NMZ (Na^+^‐Mg^+^‐Zn^2+^) electrolyte exhibits compact and orderly variations, suggesting greater consistency and reversibility, even surpassing single‐cation Na^+^ and Mg^2+^ systems as well as other combinations.

Although wavelet coefficient plots clearly reveal visually discernible differences in multi‐cycle performance among individual cations and their combinations, the quantitative assessment of the underlying statistical trends has yet to be established. To enhance objectivity and reproducibility, we extracted the within‐cycle extrema of each component at each scale *j* and cycle window *W_i_* to quantitatively characterize the evolution of critical points and establish a basis for constructing unified feature vectors for automated modeling. Specifically, the extrema $E_{\tau ,\eta }^{\left(i\right)}\left(j\right)$ are defined as:

(4) $$E_{\tau ,\max}^{\left(i\right)}\left(j\right)=\max_{t\in W_{i}}X_{\tau ,j}\left(t\right),\;E_{\tau ,\min}^{\left(i\right)}\left(j\right)=\min_{t\in W_{i}}X_{\tau ,j}\left(t\right)$$

where η ∈ max , min  denotes the extrema type.

The evolution of maxima and minima across 14 cations conditions (single or combined) over 9 cycles within approximately 60 h is presented in Figure 2b and Figure S3a. The tri‐cation combinations NMZ, NKZ, and NRZ exhibit stable approximation extrema (both maxima and minima), reflecting consistent overall trends and enhanced stability during de‐insertion relative to the single‐cation systems (Li^+^, Na^+^, and Mg^2+^). In contrast, the electrolyte systems containing Sr^2+^, Ba^2+^, and LNZ exhibit abrupt fluctuations during the initial cycles, reflecting inferior electrochemical stability and poor incompatibility. For the detailed components, examination of most cations shows a progressive decline in peak magnitudes, representing attenuation of short‑term electrochemical perturbations and enhanced reversibility. Systems containing Rb^+^ and Ca^2+^ display pronounced extrema spikes across multiple scales, strongly indicating irreversible behavior, primarily attributable to the larger ionic radii of Rb^+^ and Ca^2+^, which induce structural cracking, consistent with the DFT results.

Additionally, to precisely capture dynamic changes across cycles, inter‐cycle differences of extrema were calculated as follows:

(5) $$\Delta_{\tau ,\eta }^{\left(i\right)}\left(j\right)=E_{\tau ,\eta }^{\left(i+1\right)}\left(j\right)-E_{\tau ,\eta }^{\left(i\right)}\left(j\right),\;i=1,\text{…},N-1$$

Accordingly, for each (τ, η, *j*), the sequence $\Delta_{\tau ,\eta }^{\left(i\right)}\left(j\right)i=1^{N-1}$ is obtained, where $\Delta_{\tau ,\eta }^{\left(i\right)}\left(j\right)$ denotes the magnitude of the change in extrema between adjacent cycles. The maximum differences $\Delta_{\tau ,\max}^{\left(i\right)}\left(j\right)$ typically reflect high‐frequency spikes induced by abnormal discharge events or side reactions, whereas the minimum differences $\Delta_{\tau ,\min}^{\left(i\right)}\left(j\right)$ capture persistent negative shifts that indicate local structural failure. Pronounced cycle‐to‐cycle fluctuations in these difference sequences are indicative of potential electrochemical degradation and intrinsic structural damage.

On this basis, the cross‐cycle difference sequence ${\left\{\Delta_{X,j,g}^{\left(c\right)}\right\}}_{c=1}^{C-1}$ was constructed, and its distribution was analyzed using box‐plot visualization. To quantitatively characterize fluctuation patterns and systematically compare stability and reversibility across cation conditions, we extract three robust statistics: the median $\Delta_{\tau ,\eta }^{med}\left(j\right)$ is used to evaluate the typical fluctuation level; the interquartile range (IQR) $\Delta_{\tau ,\eta }^{IQR}\left(j\right)$ to quantify fluctuation amplitude, and the outlier count $\Delta_{\tau ,\eta }^{out}\left(j\right)$ to characterize the frequency of extreme fluctuations. Box plot indices and statistical features facilitate the identification of both high‐quality candidates and anomalous behavior, as evidenced by consistent median values and minimal inter‐cycle variability. In this framework, the median, IQR, and outlier count were selected as core descriptors because they map directly onto key physical dimensions of electrochemical stability. The median anchors the thermodynamic baseline of the system. The IQR quantifies inter‐cycle consistency. The outlier count serves as a sensitive precursor indicator, effectively capturing stochastic electrochemical instabilities and transient kinetic perturbations that are typically obscured in conventional macroscopic metrics.

As a result, the Na^+^, Mg^2+^, NMZ, and NRZ systems present tightly clustered box plots with minimal variation in both maxima and minima (Figure 2c and Figure S3b), indicating stable cycling behavior and high reversibility. In contrast, the pristine electrolyte and cations Li^+^, Ca^2+^, Sr^2+^, Ba^2+^, LMZ, and LNZ show large fluctuations, reflected by wide box plots and numerous outliers, suggesting significant trend drift (instability) and potential periodic anomalies (irreversibility) during cycling. Moreover, these cations display pronounced fluctuations at the detail level D_1, as evidenced by enlarged box plots and extended whiskers, reflecting the high variability and localized structural deterioration. The K^+^, Rb^+^, LMZ, and NKZ cationic systems exhibit comparatively amplified response in the detail components relative to the approximation extrema, meaning the strong micro‐scale influence of cations intercalation, as corroborated by DFT modeling. The pristine and cations Ca^2+^‐, Sr^2+^‐, and Ba^2+^‐based electrolytes continue to show pronounced fluctuations at detail level D_3. Notably, the NMZ electrolyte shows stable behavior across all detail scales (D_1 to D_3), demonstrating its potential as a predictable and reliable candidate for practical applications.

**FIGURE 3:**
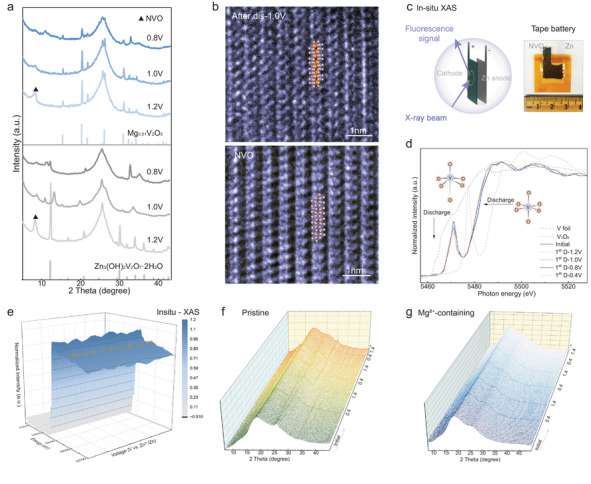
Postmortem electrode characterization and analysis. (a) Selected XRD patterns of electrodes cycled in pristine (bottom) and Mg^2+^‐containing (top) electrolytes at different discharge states. (b) High‐resolution TEM images of fresh NVO and after discharge to 1.0 V in Mg^2+^‐containing electrolyte. (c) Schematic illustration of XAS using tape‐type batteries. (d) Normalized V *K*‐edge XANES spectra of NVO during the first discharge process. (e) In situ XANES spectra over the initial two cycles. In situ XRD performance of electrodes cycled in pristine (f), and Mg^2+^‐containing (g) electrolytes.

Post‐mortem electrode characterization confirmed the role of cation insertion. After being discharged to 1.2 V (vs Zn^2+^/Zn) using pristine electrolyte, the cathodes emerged new XRD peaks as shown in Figure 3a, which can be indexed to zinc hydroxide vanadium hydrate Zn_3_(OH)_2_V_2_O_7_·2H_2_O (V‐LDH, PDF#87‐0417), indicating severe vanadium dissolution [20, 21]. SEM characterization shows flower‐like precipitates on the electrode surface (Figure S4a,b), which are consistent with the hexagonal and plate‐like microstructures composed of V, O, and Zn elements observed in TEM analysis (Figure S4c). The negligible Zn signal intensity observed in the cathode suggests that this process proceeds without Zn^2+^ intercalation (Figure S4d). Theoretical calculations further confirm that the insertion potential initiates at approximately 1.0 V for hydrated Zn^2+^ and at 0.8 V for single Zn^2+^ (Figure S5), consistent with the homogeneous spatial distribution of Zn signal observed in element mapping at 0.8 V (Figure S4g). After discharge below 0.8 V, plate‐like LDH deposits form on the electrode surface, as confirmed by SEM (Figure S4e,f). These deposits are devoid of V (Figure S4h) and are well‐documented parasitic precipitation products (LDH) in ZIBs. The electrode cycled in Na^+^‐containing electrolyte exhibits a relatively weak V‐LDH peak at 1.2 V that presents at 0.8 V (Figure S6). This result indicates a limited capacity to suppress vanadium dissolution, despite successful Na insertion, as shown in Figure S7. Notably, distinct Mg_0.01_V_2_O_5_ (PDF#89‐0610) diffraction peaks emerge in the NVO electrode cycled in the Mg^2+^‐containing electrolyte and remain detectable even after being discharged to 0.8 V. The high‐resolution TEM image reveals a clear transformation from a uniformly rippled structure with (110) planes to a circularly organized morphology after discharge to 1.0 V (Figure 3b), rather than the disordered arrangement observed in the pristine electrolyte (Figure S8). EDS mapping of enriched Mg distribution confirms the successful insertion of Mg^2+^ upon discharge at both 1.0 and 0.8 V (Figures S9a,b). The progressively decreasing Gibbs free energy associated with Mg^2+^ insertion further confirms its thermodynamic favorability (Figure S10), supporting structural stability and enabling Mg^2+^ to function effectively as active charge carriers. As illustrated in Figure S9c, Mg^2+^ electrochemical insertion initiates at an onset discharge voltage of 1.2 V (vs. Zn^2+^/Zn).

Using tape‐type Zn/NVO batteries, ex situ and in situ X‐ray absorption spectroscopy (XAS) measurements were conducted to investigate structural transformations during cycling (Figure 3c). After the first discharge to 1.0 V, the electrode cycled in the Mg^2+^‐containing electrolyte shows only a 0.57 eV shift toward lower photon energy (Figure 3d), in contrast with the pronounced 1.3 eV shift observed in the pristine electrolyte (Figure S11). This minimal shift indicates that Mg^2+^ insertion confers a favorable spatial configuration advantage, es evidenced by the formation of the Mg_0.01_V_2_O_5_ transition phase. Moreover, the enhanced white‐line intensity reflects an increase in the population of unoccupied V 3*d* states [22], thereby facilitating electron density redistribution and weakening V‐O hybridization. A significant increasing trend was observed above 1.0 V, indicating that the vanadium electrochemical dissolution predominantly occurs in the high‐voltage region. In contrast, the electrode cycled in the Mg^2+^‐containing electrolyte exhibits only a marginal increase in white‐line intensity.

In situ XAS was conducted over the first two cycles to provide a more comprehensive, real‐time insight. As shown in Figure 3e, the V *K*‐edge XANES spectra with Mg^2+^‐containing electrolyte display well‐defined, periodic white‐line intensity modulations, indicating high structure reversibility of the local vanadium coordination environment during cation deintercalation relative to that of the pristine electrolyte (Figure S12). This suggests that Mg^2+^ insertion exerts a minimal influence on the core V valence states and electronic configuration, avoiding the disruption of the VO_6_ octahedra that comprise the layered framework. XPS further confirms the minimal chemical shift in vanadium valence states in the Mg^2+^‐containing electrolyte (Figure S13). In situ XRD characterizations over three cycles further confirm that the severe vanadium dissolution in the pristine electrolyte, as evidenced by the two distinct and progressively intensified peaks at 12.29° and 30.1°, well‐indexing to the (001) and (002) planes of V‐LDH (Figure 3f and Figure S14a), consistent with the ex situ XRD results obtained from coin batteries. In contrast, the Mg^2+^‐containing electrolyte effectively suppresses vanadium dissolution, as evidenced by the absence of V‐LDH signal (Figure 3g and Figure S14b).

To assess the influence of the Zn^2+^ solvated structure, the electrolyte configuration was investigated using molecular dynamics (MD) simulations. Compared with the pristine electrolyte, the MgZn‐1.0 electrolyte (2.0 m ZnSO_4_ + 1.0 m MgSO_4_) exhibits an increased population of contact ion pairs (CIPs), rising from 30.93% to 40.10%, accompanied by a decrease in solvent‐separated ion pairs (SSIPs) from 57.03% to 39.14% (Figure S15a). This observation suggests that an increased population of SO_4_^2−^ anions is involved in the primary Zn^2+^ solvation shell, promoting the desolvation process through a reduction in the overall hydration energy, consistent with conditions observed in crowded or highly concentrated electrolytes [23, 24]. X‐ray absorption spectroscopy (XAS) was employed to directly probe the actual Zn^2+^ solvation structure in the electrolyte. According to the Zn *K*‐edge X‐ray absorption near‐edge structure (XANES) spectra, Zn in the MgZn‐based electrolyte exhibits an increased effective positive charge with increasing Mg^2+^ concentration (Figure S15b). This behavior arises from the electron‐accepting nature of the co‐cation Mg^2+^ within the emerging solvation structure, which withdraws electron density from the coordinating O atoms of H_2_O. Consequently, the electron density surrounding Zn^2+^ decreases, thereby enhancing the ionic character of Zn‐O bonds, as evidenced by the intensified white‐line feature.

The detailed coordination information obtained from Fourier‐transformed extended X‐ray absorption fine structure (FT‐EXAFS) spectra shows that the MgZn electrolytes exhibit negligible changes near 1.6 Å, whereas a pronounced peak shift is observed around 2.2 Å as Mg concentration increases from 0.2 m to saturation (Figure S15c). Wavelet transform analysis of EXAFS spectra confirms the presence of Zn‐O coordination in both pristine (Figure S15d) and MgZn‐1.0 electrolytes (Figure S15e) [25, 26], indicating that Mg^2+^ interacts with Zn^2+^ through oxygen atoms without directly entering the first solvation shell of Zn^2+^. The high electron density signal at approximately 3.0 Å is slightly perturbed with increasing Mg^2+^ concentration, which is attributed to long‐range coordination effects. The presence of Mg^2+^ reduces the coordination symmetry of hydrated Zn^2+^, thereby facilitating its desolvation. The shift toward higher frequencies in the 3250–3304 cm^−1^ range of the Raman spectra (Figure S15f) is consistent with the decrease of free water molecules (Figure S16) and the increased presence of [HOH‐OSO_3_^2−^] ligands near 3450 cm^−1^ shift [27, 28, 29, 30]. Notably, the first solvation shell in the 3 m ZnSO_4_ (3 m) electrolyte is less pronounced than that in MgZn‐1.0 (Figure S17a), while the reduced white‐line intensity indicates much weaker polarized and sluggish Zn^2+^ species within the solvation structure. In the triple‐cation NMZ electrolyte, the heightened white‐line intensity supports the conclusion that Na^+^ incorporation significantly reduces the electron density distributions around Zn^2+^ (Figure S17b), exceeding the effect observed in the MgZn binary system.

Zn plating/stripping behavior was evaluated using symmetric Zn/Zn cells. After three CV cycles at 0.1 mV s^−1^, the Zn electrode cycled in the pristine electrolyte exhibits a distinctly irregular surface morphology with heterogeneous grain distribution (Figure S18a). In contrast, the electrode cycled in the MgZn‐1.0 electrolyte shows a more homogenous morphology with uniformly oriented grains (Figure S18b). XRD confirms a preferential orientation toward the Zn(101) facet in the MgZn electrolytes, as evidenced by the increased intensity ratio of *I_(101)/(002)_* as shown in Figure S18c, which rises from 1.50 at 0.2 m to 5.72 in the saturated state (MgZn‐Sta). This observation agrees well with the theoretical crystal models (Figure S18d). Among the concerned surfaces (Figure S18e), Mg^2+^ exhibits the highest adsorption energy on the Zn(002) facet (−2.42 eV), followed by Zn(100) facet (−1.57 eV), and Zn(101) facet (−1.39 eV). These preferential absorptions of Mg^2+^ effectively hinder the sequential deposition of Zn^2+^, thereby guiding selective deposition and suppressing dendrite growth.

The desolvation capability of hydrated Zn^2+^ at the electrode interface was evaluated by activation energy (*Ea*) based on Arrhenius' law of equations (Figure S18g). The MgZn‐1.0 electrolyte exhibits a lower activation energy (48.4 kJ mol^−1^) than the pristine electrolyte (51.0 kJ mol^−1^), predicting that the hydrated Zn^2+^ undergoes interfacial desolvation more readily, due to the reduced coordination energy of hydrated Zn^2+^ discussed earlier. The dynamic Zn plating behavior was analyzed using 2D grazing‐incidence X‐ray diffraction (GIXD). Fresh Zn exhibits typical Debye–Scherrer rings at 2*θ* angles of 36.29°, 43.22°, and 54.32°, which are indexed to the (002), (100), and (101) crystallographic planes of metallic Zn, respectively (Figure S18h). After the initial cycle, the diffraction spots become uniformly distributed along the (101) ring, suggesting an isotropic orientation growth and homogeneous Zn deposition at the beginning. After the third cycle, the diffraction intensity is markedly enhanced on the (101) reflection while substantially suppressed on the (002) reflection, confirming preferential crystallographic growth along the (101) orientation. The integrated GIXD patterns corroborate the observed texture transition (Figure S19a) and are further validated using a different type of Zn anode (Figure S19b).

**FIGURE 4:**
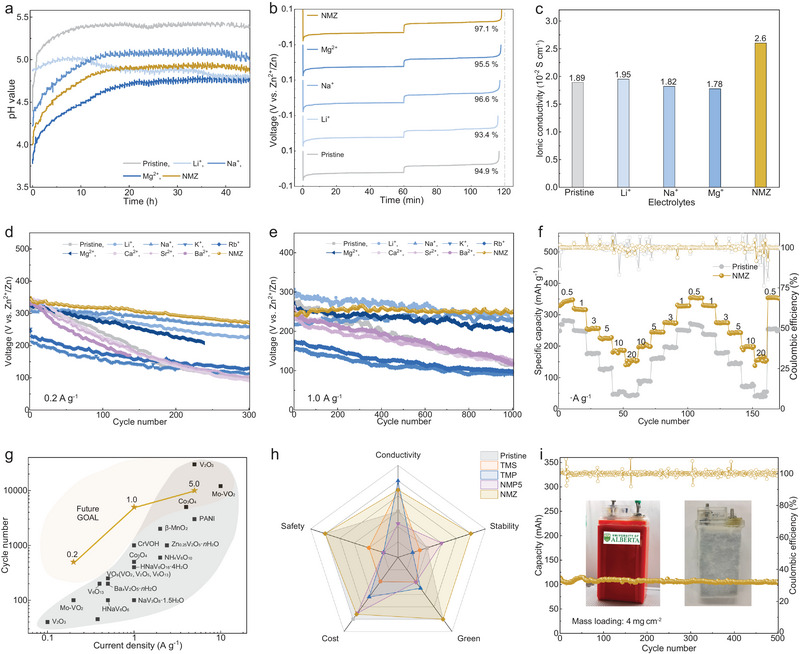
Electrochemical performance and comparison. (a) In situ pH evolution of symmetric Zn/Zn cells. (b) Deposition/stripping curves of Cu/Zn cells. (c) Ionic conductivity of the electrolytes. Long‐term cycling performance of Zn/VO batteries at current densities of 0.2 A g^−1^ (d) and 1.0 A g^−1^ (e). (f) Rate performance of Zn/NVO batteries cycled in the pristine and tri‐cation NMZ electrolytes. (g) Comparison of this work with previously reported references in terms of current density and cycle number: *β*‐MnO_2_ [33], *α*‐MnO_2_ [34], Co_3_O_4_ [35], Zn_0.25_V_2_O_5_·H_2_O [5], CrVOH [36], VO*s* (VO_2_, V_2_O_5_, V_6_O_13_) [37], HNaV_6_O_6_ [38], and Mo‐VO_2_ [39]. (h) Radar chart comparison across five key criteria relative to reported TMS [40], TMP [41], and NMP5 electrolytes [42]. (i) Cycling performance of the five‐layer prismatic battery with a total active material mass of 720 mg using the NMZ electrolyte, accompanied by post‐cycling internal images.

The in situ pH evolution of Zn/Zn symmetric cells was monitored to analyze the electrochemical reversibility and side reactions during the Zn plating/stripping process. In the pristine electrolyte, as shown in Figure 4a, the pH value increased markedly from 4.6 to 5.4 within just 45 h (45 cycles) at a plating capacity of 1 mAh cm^−2^, revealing severe hydrogen evolution reactions (HER). Owing to the inherently alkaline nature of Li_2_SO_4_, the Li^+^‐containing electrolyte delivers a higher initial pH of 4.87 and maintains pH stability throughout cycling. The Na^+^‐containing electrolyte exhibits a slightly lower pH than the pristine electrolyte and stabilizes at around 5.04, due to the electrostatic shielding effect of Na^+^ [31, 32]. The Mg^2+^‐containing electrolyte exhibits the most stable pH evolution, consistently maintaining a low pH value of 4.77, whereas the NMZ electrolyte shows intermediate pH behavior (4.90) with a balance between the effects of Na^+^ and Mg^2+^. In Cu/Zn cells, the NMZ electrolyte achieves a coulombic efficiency of 97.1% (Figure 4b), surpassing those of the single‐cation and pristine electrolytes, and displays the highest ionic conductivity of 2.60 × 10^−2^ mS cm^−1^ (Figure 4c).

Guided by the preceding analysis, we then evaluated the data‐driven electrolytes to investigate the effects of cation on the reversibility and long‐term stability of Zn/NVO batteries. Compared with the pristine electrolyte, the incorporation of Li^+^, Na^+^, and Mg^2+^ cations enhances electrochemical stability at current densities of 0.2 A g^−1^ (Figure 4d), 1.0 A g^−1^ (Figure 4e), and 5.0 A g^−1^ (Figure S20). The addition of Ca^2+^, Sr^2+^, and Ba^2+^ exerts a detrimental effect. Notably, the NMZ tri‐cationic electrolyte delivers the most robust cycling stability, sustaining 500 cycles at 0.2 A g^−1^ and 5000 cycles at 1.0 A g^−1^ with nearly 100% capacity retention after 1000 cycles. In situ XRD measurement of the NMZ electrolyte confirms the absence of V‐LDH peaks, indicating negligible vanadium dissolution (Figure S21). Moreover, the tri‐cation NMZ electrolyte delivers outstanding rate performance, maintaining a capacity of ~150.5 mAh g^−1^ even at 20 A g^−1^, compared with only ~43.2 mAh g^−1^ for the pristine electrolyte (Figure 4f). The as‐predicted NMZ electrolyte is further validated in Zn/VO batteries (Figure S22). Remarkably, the tri‐cationic NMZ electrolyte achieves record‐breaking stability compared with previously reported ZIBs (Figure 4g). It also demonstrates well‐balanced performance across five key criteria: conductivity, stability, environmental friendliness, cost‐efficiency, and safety, as reflected by the nearly full radar chart compared with previous reported studies (Figure 4h and Table S2). To demonstrate scalability, we fabricated a large full cell with a capacity of 0.1 Ah, composed of a five‐layer electrode, which delivers exceptional cycling stability with no sign of dissolution after cycling (Figure 4i).

**FIGURE 5:**
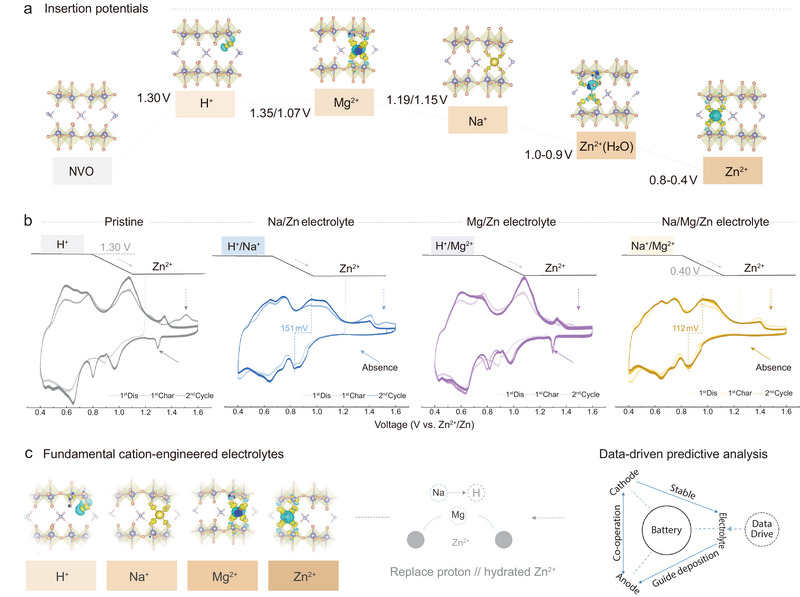
Fundamental mechanisms and analysis insights. (a) Insertion potentials of ions into the NVO cathode. (b) CV curves of Zn/NVO battery cycled in pristine, Na^+^‐ and Mg^2+^‐containing, and NMZ electrolytes at 0.1 mV s^−1^. (c) Schematic illustration of the fundamental understanding of cation‐engineered electrolytes through a data‐driven approach.

Theoretical calculations were conducted to further elucidate the underlying mechanism of cation insertion. The calculated Na^+^ insertion potentials of 1.15 and 1.19 V (Figure 5a and Figure S23) are consistent with the experimental observations, whereas Mg^2+^ inserts at 1.35 and 1.07 V. Compared with the pristine electrolyte, the Na^+^‐containing electrolyte markedly suppresses the irreversible redox activity above 1.2 V and effectively eliminates the redox peak at 1.30/1.35 V (Figure 5b), which is predominantly attributed to proton‐related insertion reactions that induce vanadium dissolution and subsequent V‐LDH formation on the electrode surface. The Mg^2^^+^‐containing electrolyte also exhibits pronounced suppression of redox activity above 1.4 V and shows a more reversible CV profile over two consecutive cycles. Given the Mg insertion at 1.07 V and its nearly identical insertion potential to that of hydrated Zn^2+^, the improved reversibility likely originates from the replacement of partially hydrated Zn^2+^ by the single Mg^2+^. This is supported by the distinct crystalline distortions induced by hydrated Zn^2+^(H_2_O) insertion as shown at 1.01 V (Figure S5), which further corroborate its contribution to vanadium dissolution. Notably, the NMZ electrolyte shows a synergistic effect between Na^+^ and Mg^2+^, as evidenced by the absence of redox peaks at 1.30/1.35 V and the negligible irreversible electrochemical features above 1.4 V. The decreased potential difference (△E) of 112 mV, compared to 151 mV in the Na‐based electrolyte, confirms that Mg^2+^ insertion contributes positively to Zn^2+^ insertion dynamics. As summarized in Figure 5c, data‐driven‐assisted cation engineering in the electrolyte triggers a potential‐driven sequential insertion of Na^+^, Mg^2+^, and Zn^2+^. This electrolyte predominantly suppresses the deteriorate proton or hydrated‐proton insertion above 1.30 V and hydrated Zn^2+^ insertion near 1.0 V (vs. Zn^2+^/Zn).

## 3 Discussion

In this work, we developed a cation‐engineered electrolyte guided by density functional theory (DFT) calculations and a multi‐scale validation framework to address the long‐standing and persistent dissolution issues in aqueous vanadium oxide‐based ZIBs. The predicted Na^+^‐Mg^2+^‐Zn^2+^ tri‐cationic electrolyte driven by the single‐ion features enables Zn/VO*_x_* batteries to unprecedented cycling stability, sustaining 500 cycles at 0.2 A g^−1^ (1400 h) and 10,000 cycles at 5 A g^−1^. The exceptional stability can be attributed to the sequential ion‐insertion of Na^+^, Mg^2+^, and Zn^2+^, strongly suppressing the deteriorated hydrated Zn^2+^ insertion at ~1.0 V and proton insertion near or above 1.3 V. This work not only provides valuable data‐driven insight into ion‐engineering electrochemistry for regulating nanoscale insertion stability, but also unveils critical ion‐related factors that are often overlooked in battery development. Given that these features originate from multi‐scale statistical “health” characterization of CV signals, this framework possesses strong physical generalizability and can be readily transferred to other electrochemical systems. Its application to other systems requires calibration of the decomposition levels to match the specific time‐frequency windows of individual redox processes. Such calibration is essential, as these processes are inherently modulated by complex anion‐solvent interactions and in situ reaction chemistries. Therefore, more comprehensive multi‐parameter analyses under broader experimental conditions should be considered in follow‐up studies.

## Conflicts of Interest

The authors declare no conflicts of interest.

## Supporting information




**Supporting File**: adma73013‐sup‐0001‐SuppMat.docx.

## Data Availability

The data that support the findings of this study are available from the corresponding author upon reasonable request.;

## References

[1] L. E. Blanc , D. Kundu , and L. F. Nazar , “Scientific Challenges for the Implementation of Zn‐Ion Batteries,” Joule 4 (2020): 771–799, 10.1016/j.joule.2020.03.002.

[2] F. Xie , H. Li , X. Wang , et al., “Mechanism for Zincophilic Sites on Zinc‐Metal Anode Hosts in Aqueous Batteries,” Advanced Energy Materials 11 (2021): 2003419, 10.1002/aenm.202003419.

[3] R. Ling , S. Zhao , C. Meng , W. Wang , C. Yang , and W. Qi , “Vertical VOPO_4_·2H_2_O Nanosheets with Richly Exposed (200) Plane Enable Fast‐Kinetics High‐Mass‐Loading Cathodes for Zn‐Ion Batteries,” Small 20 (2024): 2404089. 10.1002/smll.202404089 39036855

[4] L. He , C. Lin , L. Zeng , et al., “Synergistic Regulation of Anode and Cathode Interphases via an Alum Electrolyte Additive for High‐Performance Aqueous Zinc‐Vanadium Batteries,” Angewandte Chemie, International Edition 64 (2024): 202415221. 10.1002/anie.202415221 39324946

[5] D. Kundu , B. D. Adams , V. Duffort , S. H. Vajargah , and L. F. Nazar , “A High‐Capacity and Long‐Life Aqueous Rechargeable Zinc Battery Using a Metal Oxide Intercalation Cathode,” Nature Energy 1 (2016): 16119, 10.1038/nenergy.2016.119.

[6] L. Wang , K.‐W. Huang , J. Chen , and J. Zheng , “Ultralong Cycle Stability of Aqueous Zinc‐Ion Batteries with Zinc Vanadium Oxide Cathodes,” Science Advances 5 (2019): 4279, 10.1126/sciadv.aax4279. PMC6984968 32047853

[7] X. Dou , X. Xie , S. Liang , and G. Fang , “Low‐Current‐Density Stability of Vanadium‐Based Cathodes for Aqueous Zinc‐Ion Batteries,” Science Bulletin 69 (2024): 833–845, 10.1016/j.scib.2024.01.029. 38302333

[8] Y. Yang , Y. Tang , G. Fang , et al., “Li^+^ intercalated V_2_O_5_ *n*H_2_O with Enlarged Layer Spacing and Fast Ion Diffusion as an Aqueous Zinc‐Ion Battery Cathode,” Energy & Environmental Science 11 (2018): 3157–3162, 10.1039/C8EE01651H.

[9] P. He , G. Zhang , X. Liao , et al., “Sodium Ion Stabilized Vanadium Oxide Nanowire Cathode for High‐Performance Zinc‐Ion Batteries,” Advanced Energy Materials 8 (2018): 1702463, 10.1002/aenm.201702463.

[10] B. Tang , J. Zhou , G. Fang , et al., “Engineering the Interplanar Spacing of Ammonium Vanadates as a High‐Performance Aqueous Zinc‐Ion Battery Cathode,” Journal of Materials Chemistry A 7 (2019): 940–945, 10.1039/C8TA09338E.

[11] C. Xia , J. Guo , P. Li , X. Zhang , and H. N. Alshareef , “Highly Stable Aqueous Zinc‐Ion Storage Using a Layered Calcium Vanadium Oxide Bronze Cathode,” Angewandte Chemie International Edition 57 (2018): 3943–3948, 10.1002/anie.201713291. 29432667

[12] M. Yan , P. He , Y. Chen , et al., “Water‐Lubricated Intercalation in V_2_O_5_ ·nH_2_O for High‐Capacity and High‐Rate Aqueous Rechargeable Zinc Batteries,” Advanced Materials 30 (2018): 1703725, 10.1002/adma.201703725. 29131432

[13] S. Liu , H. Zhu , B. Zhang , et al., “Tuning the Kinetics of Zinc‐Ion Insertion/Extraction in V_2_O_5_ by In Situ Polyaniline Intercalation Enables Improved Aqueous Zinc‐Ion Storage Performance,” Advanced Materials 32 (2020): 2001113, 10.1002/adma.202001113. 32431024

[14] X. Ma , X. Cao , M. Yao , et al., “Organic–Inorganic Hybrid Cathode With Dual Energy‐Storage Mechanism for Ultrahigh‐Rate and Ultralong‐Life Aqueous Zinc‐Ion Batteries,” Advanced Materials 34 (2021): 2105452, 10.1002/adma.202105452. 34786778

[15] Y. Lu , T. Zhu , W. van den Bergh , M. Stefik , and K. Huang , “A High Performing Zn‐Ion Battery Cathode Enabled by In Situ Transformation of V_2_O_5_ Atomic Layers,” Angewandte Chemie International Edition 59 (2020): 17004–17011, 10.1002/anie.202006171. 32568438

[16] R. Sinha , X. Xie , Y. Yang , et al., “Failure Mechanisms and Strategies for Vanadium Oxide‐Based Cathode in Aqueous Zinc Batteries,” Advanced Energy Materials 15 (2025): 2404815, 10.1002/aenm.202404815.

[17] D. Chao , W. Zhou , F. Xie , et al., “Roadmap for Advanced Aqueous Batteries: from Design of Materials to Applications,” Science Advances 6 (2020): 4098. 10.1126/sciadv.aba4098 PMC7244306 32494749

[18] C. Li , A. Wang , L. Xie , et al., “Emerging Alkali Metal Ion (Li^+^, Na^+^, K^+^ and Rb^+^) doped Perovskite Films for Efficient Solar Cells: Recent Advances and Prospects,” Journal of Materials Chemistry A 7 (2019): 24150–24163, 10.1039/C9TA08130E.

[19] Z. Xing , G. Xu , J. Han , et al., “Facing the Capacity Fading of Vanadium‐Based Zinc‐Ion Batteries,” Trends in Chemistry 5 (2023): 380–392, 10.1016/j.trechm.2023.02.008.

[20] B. Zhang , L. Qin , Y. Fang , et al., “Tuning Zn^2+^ Coordination Tunnel by Hierarchical Gel Electrolyte for Dendrite‐Free Zinc Anode,” Science Bulletin 67 (2022): 955–962, 10.1016/j.scib.2022.01.027. 36546030

[21] Y. Fang , X. Xie , B. Zhang , et al., “Regulating Zinc Deposition Behaviors by the Conditioner of PAN Separator for Zinc‐Ion Batteries,” Advanced Functional Materials 32 (2021): 2109671, 10.1002/adfm.202109671.

[22] D. H. Pearson , C. C. Ahn , and B. Fultz , “White Lines and d ‐electron Occupancies for the 3d and 4d Transition Metals,” Physical Review B 47 (1993): 8471–8478, 10.1103/PhysRevB.47.8471. 10004883

[23] J. Xie , Z. Liang , and Y.‐C. Lu , “Molecular Crowding Electrolytes for High‐Voltage Aqueous Batteries,” Nature Materials 19 (2020): 1006. 32313263 10.1038/s41563-020-0667-y

[24] Z. Chang , Y. Qiao , H. Deng , H. Yang , P. He , and H. Zhou , “A Liquid Electrolyte With De‐Solvated Lithium Ions for Lithium‐Metal Battery,” Joule 4 (2020): 1776–1789, 10.1016/j.joule.2020.06.011.

[25] X. Xie , S. Liang , J. Gao , et al., “Manipulating the Ion‐Transfer Kinetics and Interface Stability for High‐Performance Zinc Metal Anodes,” Energy & Environmental Science 13 (2020): 503–510, 10.1039/C9EE03545A.

[26] X. Wang , B. Liu , Z. Xu , et al., “Characterization Techniques for Probing the Electrolyte Solvation Structures of Aqueous Zinc Metal Batteries,” Advanced Energy Materials 15 (2025): 2405253, 10.1002/aenm.202405253.

[27] H. Yang , Y. Qiao , Z. Chang , H. Deng , P. He , and H. Zhou , “A Metal–Organic Framework as a Multifunctional Ionic Sieve Membrane for Long‐Life Aqueous Zinc–Iodide Batteries,” Advanced Materials 32 (2020): 2004240, 10.1002/adma.202004240. 32797719

[28] P. Wang , X. Xie , Z. Xing , et al., “Mechanistic Insights of Mg^2+^ ‐Electrolyte Additive for High‐Energy and Long‐Life Zinc‐Ion Hybrid Capacitors,” Advanced Energy Materials 11 (2021): 2101158, 10.1002/aenm.202101158.

[29] Y. Zhang , G. Wan , N. H. C. Lewis , et al., “Water or Anion? Uncovering the Zn^2+^ Solvation Environment in Mixed Zn(TFSI)_2_ and LiTFSI Water‐in‐Salt Electrolytes,” ACS Energy Letters 6 (2021): 3458–3463, 10.1021/acsenergylett.1c01624.

[30] G. R. Pastel , T. P. Pollard , Q. Liu , et al., “Designing Interphases for Highly Reversible Aqueous Zinc Batteries,” Joule 8 (2024): 1050–1062, 10.1016/j.joule.2024.02.002.

[31] F. Wan , L. Zhang , X. Dai , X. Wang , Z. Niu , and J. Chen , “Aqueous Rechargeable Zinc/Sodium Vanadate Batteries with Enhanced Performance from Simultaneous Insertion of Dual Carriers,” Nature Communications 9 (2018): 1656, 10.1038/s41467-018-04060-8. PMC5916908 29695711

[32] Y. Xu , J. Zhu , J. Feng , et al., “A Rechargeable Aqueous Zinc/Sodium Manganese Oxides Battery with Robust Performance Enabled by Na_2_SO_4_ Electrolyte Additive,” Energy Storage Materials 38 (2021): 299–308, 10.1016/j.ensm.2021.03.019.

[33] N. Zhang , F. Cheng , J. Liu , et al., “Rechargeable Aqueous Zinc‐Manganese Dioxide Batteries with High Energy and Power Densities,” Nature Communications 8 (2017): 405, 10.1038/s41467-017-00467-x. PMC5581336 28864823

[34] H. Pan , Y. Shao , P. Yan , et al., “Reversible Aqueous Zinc/Manganese Oxide Energy Storage from Conversion Reactions,” Nature Energy 1 (2016): 16039, 10.1038/nenergy.2016.39.

[35] L. Ma , S. Chen , H. Li , et al., “Initiating a Mild Aqueous Electrolyte Co_3_O_4_ /Zn Battery with 2.2 V‐High Voltage and 5000‐Cycle Lifespan by a Co( iii ) Rich‐Electrode,” Energy & Environmental Science 11 (2018): 2521–2530, 10.1039/C8EE01415A.

[36] B. Zhang , X. Han , W. Kang , and D. Sun , “Structure and Oxygen‐Defect Regulation of Hydrated Vanadium Oxide for Enhanced Zinc Ion Storage via Interlayer Doping Strategy,” Nano Research 16 (2023): 6094–6103, 10.1007/s12274-022-4834-0.

[37] H. Song , Y. Cui , Y. Li , et al., “Alcohol Molecule Coupling: A Universal Approach to Modulating Amorphousness in Vanadium‐Based Cathodes For High‐Rate and Durable Aqueous Zinc‐Ion Batteries,” Science Advances 11 (2025): adt7502, 10.1126/sciadv.adt7502. PMC12101504 40408486

[38] X. Guo , G. Fang , W. Zhang , et al., “Mechanistic Insights of Zn^2+^ Storage in Sodium Vanadates,” Advanced Energy Materials 8 (2018): 1801819, 10.1002/aenm.201801819.

[39] D. Zhang , Y. Yue , C. Yang , et al., “Kinetics‐Boosted and Dissolution‐Suppressed Molybdenum‐Doped vanadium dioxide for Long‐Life Zinc‐Ion batteries,” Chemical Engineering Journal 506 (2025): 160160, 10.1016/j.cej.2025.160160.

[40] Y. Zhong , X. Xie , Z. Zeng , B. Lu , G. Chen , and J. Zhou , “Triple‐function Hydrated Eutectic Electrolyte for Enhanced Aqueous Zinc Batteries,” Angewandte Chemie, International Edition 62 (2023): 202310577. 10.1002/anie.202310577 37578644

[41] W. Wang , S. Chen , X. Liao , et al., “Regulating Interfacial Reaction through Electrolyte Chemistry Enables Gradient Interphase for Low‐Temperature Zinc Metal Batteries,” Nature Communications 14 (2023): 5443, 10.1038/s41467-023-41276-9. PMC10482877 37673895

[42] T. C. Li , Y. Lim , X. L. Li , et al., “A Universal Additive Strategy to Reshape Electrolyte Solvation Structure Toward Reversible Zn Storage,” Advanced Energy Materials 12 (2022): 2103231, 10.1002/aenm.202103231.

