# Supplementary material for: Data‐Driven Cation Engineering Guides Electrolyte Design for Sustainable Aqueous Zinc Battery Chemistries
Source: Adv Mater. 2026 Apr 8;38(26):e22059. doi: 10.1002/adma.202522059 (PMC13155322; doi:10.1002/adma.202522059)
Supplement: Supplementary file 1 — Supporting File: adma73013‐sup‐0001‐SuppMat.docx. [file ADMA-38-e22059-s001.docx]

Supporting Information

**Data-Driven Cation Engineering Guides Electrolyte Design for Sustainable Aqueous Zinc Battery Chemistries**

*Xuesong Xie^1^, Yinfei Lyu^2, 3^,* *Huorong Ren^2^, Witold Pedrycz^3, *^, Yifan Li^1^, Yang Yang^1^,* *Xuehai Tan^1^, Minggang Xie^1^, Yi Guan^1^, Yuxuan Xue^1^, Ning Chen^4^, and Zhi Li^1, *^*

^1^Department of Chemical and Materials Engineering, University of Alberta, Edmonton T6G 2E8 AB, Canada;

^2^ School of Electro-mechanical Engineering, Xidian University, Xi’an 710071, China;

^3^ Department of Electrical and Computer Engineering, University of Alberta, Edmonton, T6G 2V4 AB, Canada;

^4^Hard X-Ray Micro Analysis BL, Canadian Light Source, Saskatoon, Saskatchewan S7N 2V3, Canada;

**^*^Corresponding author****.** E-mail address: wpedrycz@ualberta.ca; zhi.li@ualberta.ca

**Experimental Section**

**The synthesis of NH_4_V_4_O_10_ (NVO):** 1.17 g NH_4_VO_3_ was dissolved in 80°C deionized water until it formed a yellow solution. Then, the solid powers of 1.89 g H_2_C_2_O_4_·2H_2_O were gradually added to the solution under continuous stirring. The solution was transferred to the 50 mL hydrothermal synthesis autoclave reactor and kept in the oven at 140 °C for 48 h. The remaining products were collected and washed with deionized water three times after cooling down to room temperature. The final products were dried at 60 °C for 12 h to be used.

**The synthesis of V_2_O_5_·1.2H_2_O (VO)**: 0.6 g of commercial V_2_O_5_ powder was gradually added to an aqueous solution under continuous stirring, followed by transfer to a 50 mL hydrothermal autoclave reactor and heating at 140 °C for 48 h. The resulting product was washed according to the procedure described for NVO.

**Assembly of tape Zn/NVO batteries:** The positive NVO material, carbon black, and PVDF binder (polyvinylidene difluoride) are dissolved in organic solvent NMP (1-Methyl-2-pyrrolidinone), mixed evenly with a mass ratio of 7:2:1 and coated onto the carbon paper, finally dried at 60 ^o^C. The mass loading of cathode was approximately 1.2 mg cm^-2^. The zinc foil with a thickness of 0.1 mm (12 mm in diameter) was obtained from Weifang Purun Energy Technology Limited Corporation. In the coin cell configuration, the cathode and anode were separated by a single layer of glass fiber separator (19 mm in diameter, Whatman GF/D), and assembled into a CR-2032 type cell in air conditions with an electrolyte content of 60 μL. To ensure consistency and reproducibility in investigating ions behavior, the concentrations of sodium (Na^+^), lithium (Li^+^), magnesium (Mg^2+^) cations in the electrolyte were standardized to 1 M across all experimental groups, with the addition of a 2 M ZnSO_4_ baseline electrolyte. For other cations, due to the solubility limitations, the following concentrations were used: K^+^, 0.5 M; Rb^+^, 0.2 M; Ca^2+^, Sr^2+^, and Ba^2+^ at 0.2 M each, all with the addition of 2 M ZnSO_4_ baseline electrolyte. The pristine electrolyte was chosen as a 2 M ZnSO_4_ solution. To maintain constant anion concentration, a 3 M ZnSO_4_ electrolyte was also incorporated for evaluation and analysis. For the three-cation electrolyte systems, the concentration of each cation was maintained at 1 M. For the square battery, the electrode was prepared on the 316 stainless-steel mesh (200 mesh, Fuel Cell Store). The loading of active materials was 720 mg with a corresponding mass loading of 4 mg cm^-2^. **Characterizations:** Ex-situ powder X-ray diffraction (XRD) patterns were conducted by the Rigaku Ultima IV diffractometer using Cu *K*_α_-radiation (*λ*= 1.5418). In-situ XRD diffraction were collected at Bruker D8 Discover diffraction system equipped with Cu-source and high throughput LynxEYE 1-dimensional detector. Raman spectra were recorded on a Renishaw InVIa Raman Instrument with a laser excitation wavelength of 633 nm. X-ray photoelectron spectroscopy (XPS) spectra were acquired on a Kratos Ultra XPS Spectrometer. Scanning electron microscopy (SEM) and the corresponding energy-dispersive X-ray spectroscopy (EDS) mapping were obtained on the Hitachi S-4800 FESEM. X-ray adsorption spectroscopy (XAS) spectra were performed on the Hard X-ray Microanalysis Beamline (HXMA, 061D-1) at the Canadian Light Source.

**Electrochemical measurements:** Galvanostatic charge/discharge cycling measurements were carried out on a LAND multichannel battery test system (CT2001A, China). The cyclic voltammetry (CV) and electrochemical impedance spectroscopy (EIS) were conducted by the SP-300 electrochemical workstation (EC-lab Biologic, France). The tape batteries for in-situ XRD and XAS characterization were conducted by the Gamry redefining electrochemical measurement (Reference 3000, Germany). All electrochemical measurements were conducted using at least three independent coin cells to ensure reproducibility.

**Computational method:** The density functional theory (DFT) calculation was based on the first principles^[1]^, which were conducted by the VASP with a package file of 6.4 and a DFT method. The binding energy and electron properties were based on the generalized gradient approximation (GGA) method with Perdew-Burke-Ernzerhof (PBE) functional for the exchange-correlation term^[2]^. Projector-augmented-wave (PAW) type pseudopotentials are used to describe the ionic cores and the electronic structure^[3]^. Van der Waals interaction was taken into account at DFT-D4 and take valence electrons into account using a plane-wave basis set with a kinetic energy cutoff of 500 eV. The SCF energy convergence criteria were established at 10^-8^ eV for electronic relaxation and 0.02 eV Å^-1^ for the Hellmann–Feynman force on each atom. The Brillouin zone was sampled using a 3×9×3 k-point mesh for the ion insertions. For the energy calculations, the DFT+U formation was applied to account for strong on-site Coulombic interactions of the V 3d-electrons with U=3.25 eV.

The average intercalation voltages are calculated according to the following equation:

*V* is the average voltage for M-ion insertion, where M = Li^+^, Na^+^, Zn^2+^, or Zn^2+^·H_2_O. *E(NVO·M^x+^)* represents the total energy of the ion-inserted materials. *E(NVO)* is the energy of bulk NVO. *E (M^x+^)* is the energy of a single M atom.

**Molecular dynamics simulations**: The aqueous electrolyte systems were simulated with LAMMPS. The simulation box is of 60 x 60 x 60 Å under period boundary conditions, comprising Zn^2+^ cations, fully atomistic SO_4_^2-^ anions, and water molecules. Zinc ions were reverse as single-site divalent cations same like Magnesium ions, while sulfate anions were represented using a non-reactive all-atom model with harmonic bond and angle interactions. Water molecules were modeled using rigid three-site configurations, where O-H bond lengths and H-O angles were constrained by the SHAKE algorithm. Long-range electrostatic interactions and short-range Lennard-Jones interactions were handled using standard force-field parameters. Following a multi-step annealing and equilibration procedure, long-term production simulations were conducted under the NPT ensemble at 298 K and 1 atm. Temperature and pressure were maintained using the Nosé–Hoover thermostat and barostat with damping constants of 200 fs and 2000 fs, respectively. A time step of 2 fs was employed, followed by two consecutive NPT runs of 30 ns and 20 ns, yielding a total production time of 50 ns.

Subsequently, the production simulations were conducted by NVT procedure at 298 K, aimed at analyzing the solvation structure. The system temperature was maintained using a Nosé–Hoover thermostat with a damping constant of 200 fs, while a timestep of 2 fs was applied. A total simulation time of 50 ns was performed.

**Data-drive section:** The algorithmic framework was developed in a Python 3.8 environment, relying primarily on the PyWavelets (pywt) library for the implementation of the Discrete Wavelet Transform (DWT), integrated with NumPy, Pandas, and Matplotlib for data preprocessing and statistical visualization. For the specific implementation, the Daubechies-3 (db3) wavelet family, characterized by its compact support, was consistently selected for a 3-level decomposition (Level 3). The complete source code for this study is available on GitHub at <https://github.com/xuesong-research/WaveCV.git>

To enhance the recognition of key performance indicators, this study employs the DWT with inherent multi-resolution analysis capability, which uses a family of scaled-and-shifted wavelet functions to decompose raw CV signals into low-frequency approximation components and high-frequency detail components, thus providing a joint time-frequency characterization. The low-frequency approximation components capture the global redox behavior, while the high-frequency details that are highly sensitive to transient fluctuations and localized instabilities. This method leverages superior time-frequency localization capabilities. This allows for the identification of microscopic instability features often masked by dominant redox peaks, thereby advancing the analytical scope from mere result description to the early-stage warning of failure mechanisms.

Based on the cross-cycle difference sequence $\Delta_{\tau,\eta}^{(c)}(j)_{c=1}^{C-1}$, we further extract the following robust statistical indices:

Median provides resistance to extreme values while reflecting central tendency:

$$\Delta_{\tau,\eta}^{med}(j)=\mathrm{median} \left(\Delta_{\tau,\eta}^{(c)}(j)\right)$$

Interquartile range (IQR) captures the spread of typical fluctuations independent of distribution assumptions:

$$\Delta_{\tau,\eta}^{IQR}(j)=Q_{3} \left(\Delta_{\tau,\eta}^{(c)}(j)\right)-Q_{1} \left(\Delta_{\tau,\eta}^{(c)}(j)\right)$$

Outlier count specifically targets abnormal electrochemical events that may indicate degradation mechanisms:

$$\Delta_{\tau,\eta}^{out}(j)=|\{ \Delta_{\tau,\eta}^{(c)}(j) : \Delta_{\tau,\eta}^{(c)}(j)\notin[ Q_{1}-1.5 \Delta_{\tau,\eta}^{IQR}(j), Q_{3}+1.5 \Delta_{\tau,\eta}^{IQR}(j) ]\}|$$

The selection of these three statistics is motivated by their complementary roles in characterizing electrochemical cycling: consistent median and IQR indicate stable phases, whereas the Tukey 1.5×IQR outlier count captures the frequency of extreme fluctuations.

**Supplementary Materials**

**Figure S1.** Correlation between cation atomic number of and its bonding energy in VO-based materials.


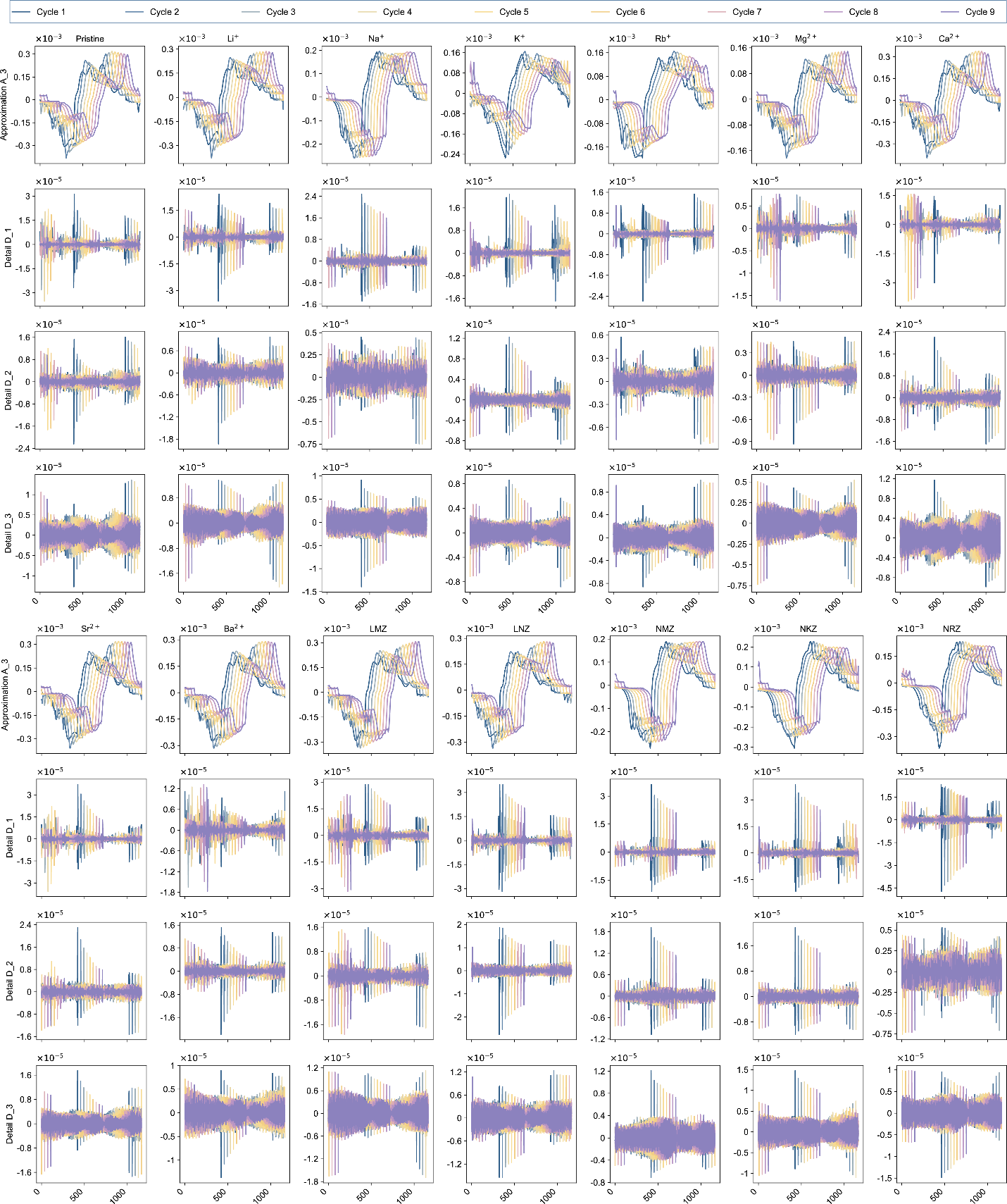


**Figure S2**. Multi-scale decomposition of CV curves at scan rate of 0.1 mV s^-1^ for different electrolyte systems.


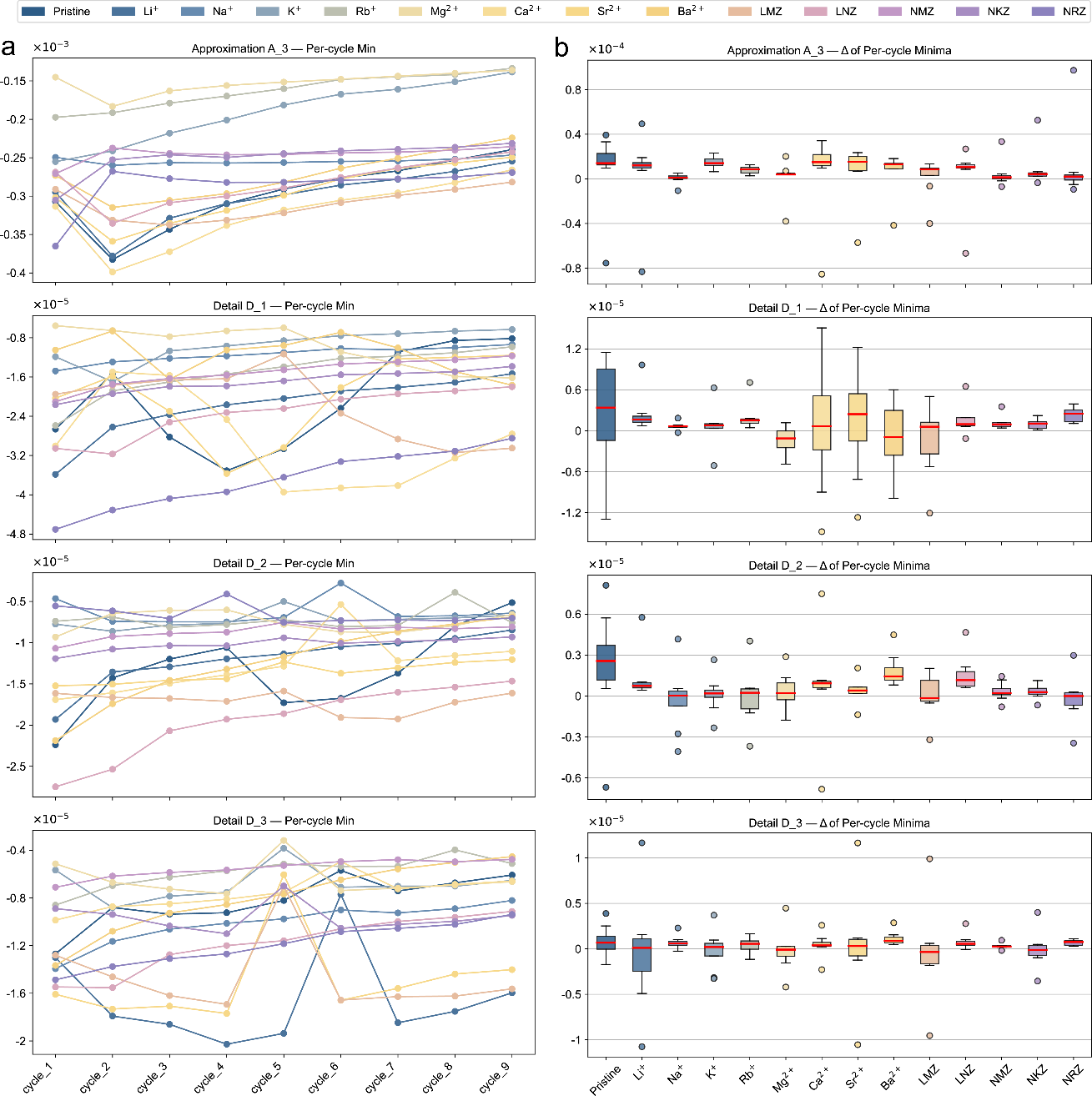


**Figure S3**. (a) Multi-scale evolution of the minimum cation insertion level and trend across successive electrochemical cycles. (b) Box-plot distribution of the maxima and cycling stability analysis for different electrolyte systems.


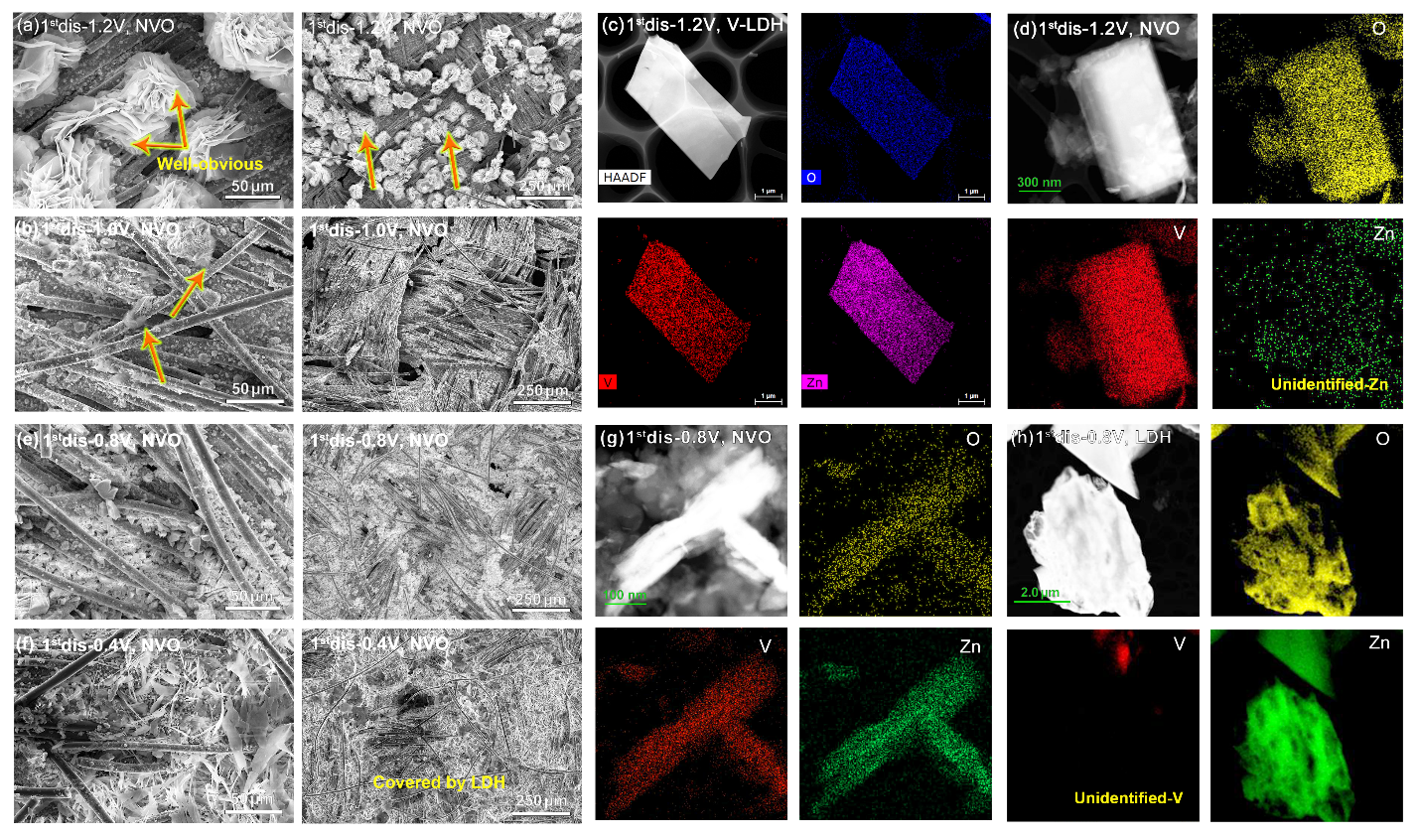


**Figure S4.** SEM images of the NVO electrodes after the first discharge of 1.2 V (a), 1.0 V (b), 0.8 V (e), and 0.4 V (f). Corresponding TEM and EDS images of parasitic products (c) and the electrode (d) at 1.2 V (*vs.* Zn^2+^/Zn), along with the electrode (g) and parasitic products (h) after discharge to 0.8 V (*vs.* Zn^2+^/Zn).


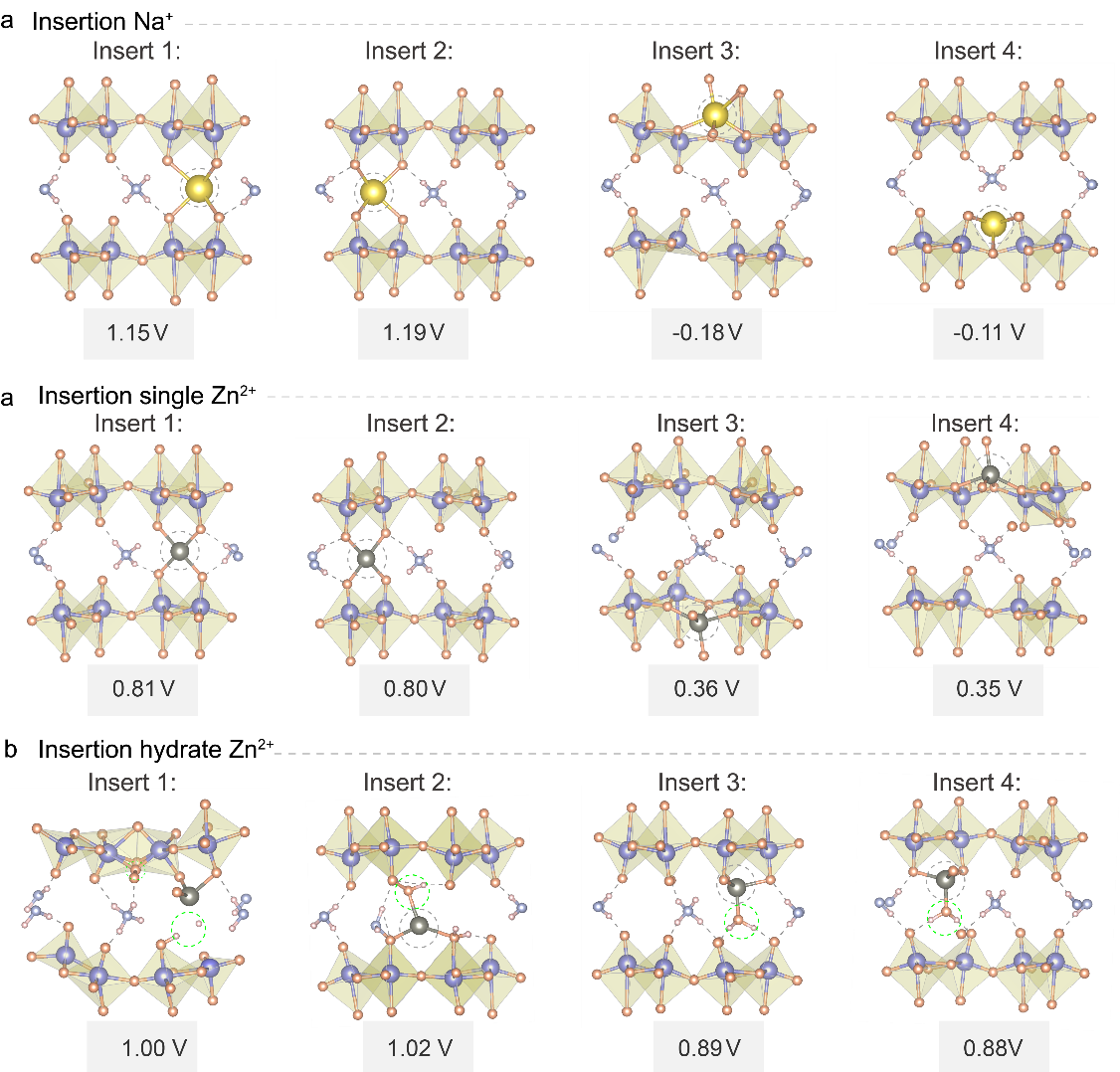


**Figure S5.** Theoretical insertion sites and voltages for single Zn^2+^ (a), and hydrated Zn^2+^(H_2_O) (b) in NVO-based materials.


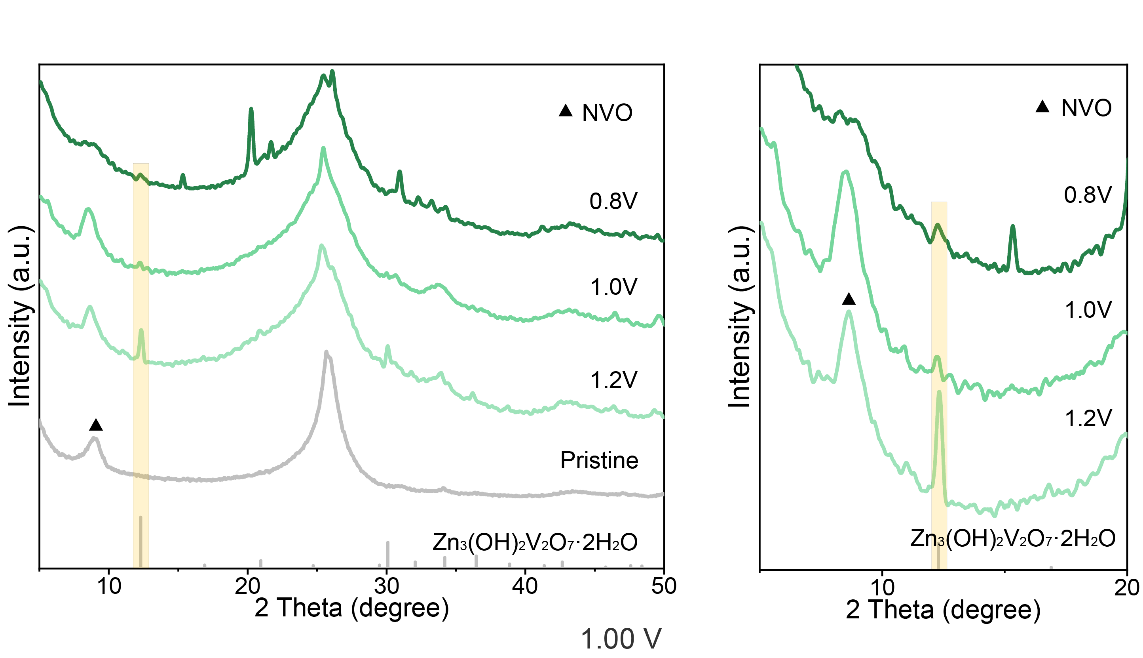


**Figure S6.** XRD patterns of NVO electrodes cycled in Na^+^-containing electrolytes during discharge process.


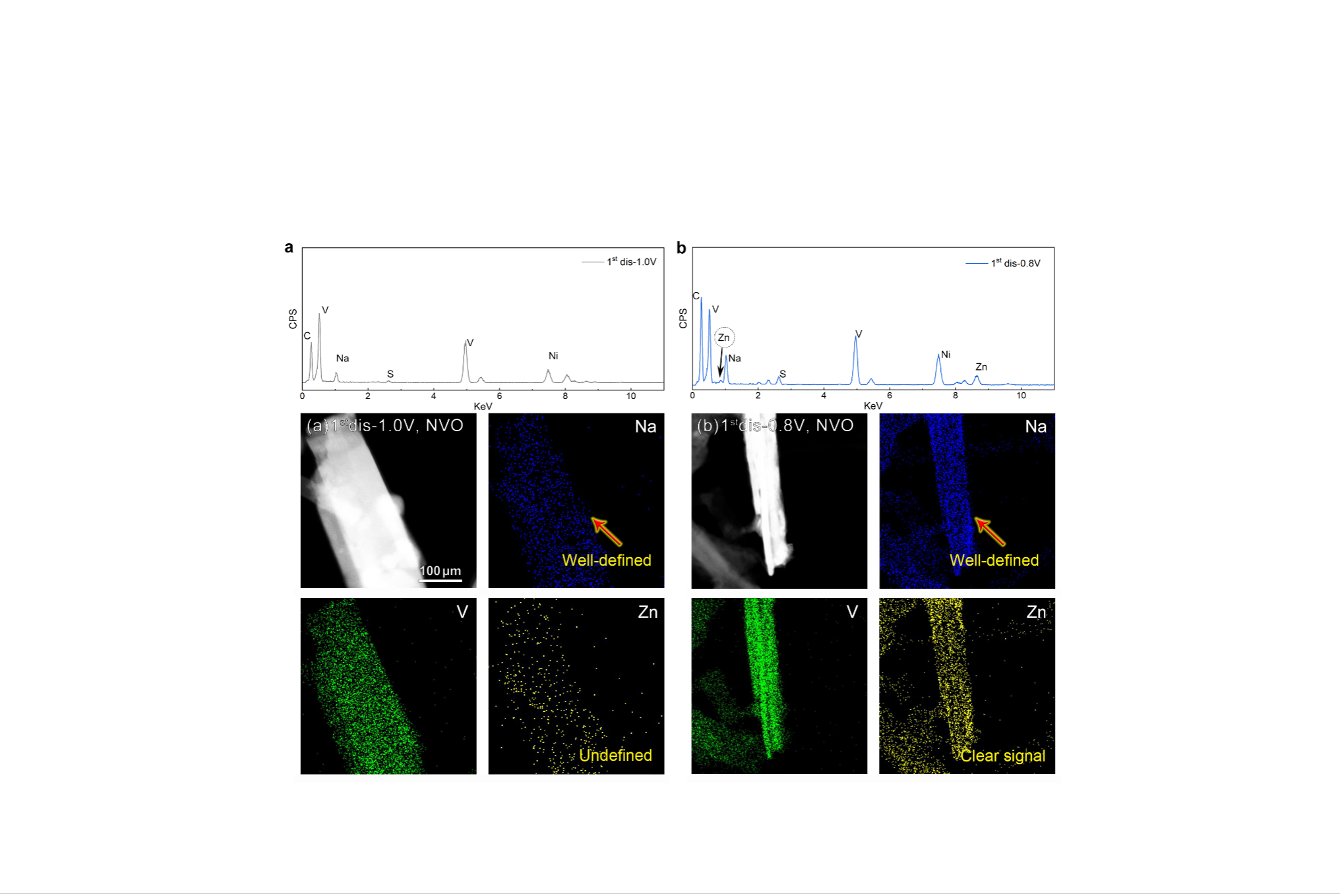


**Figure S7.** Energy-dispersive X-ray spectroscopy (EDS) elemental mapping and corresponding spectra acquired after the first discharge to 1.0 V (a), and 0.8 V (b), respectively.


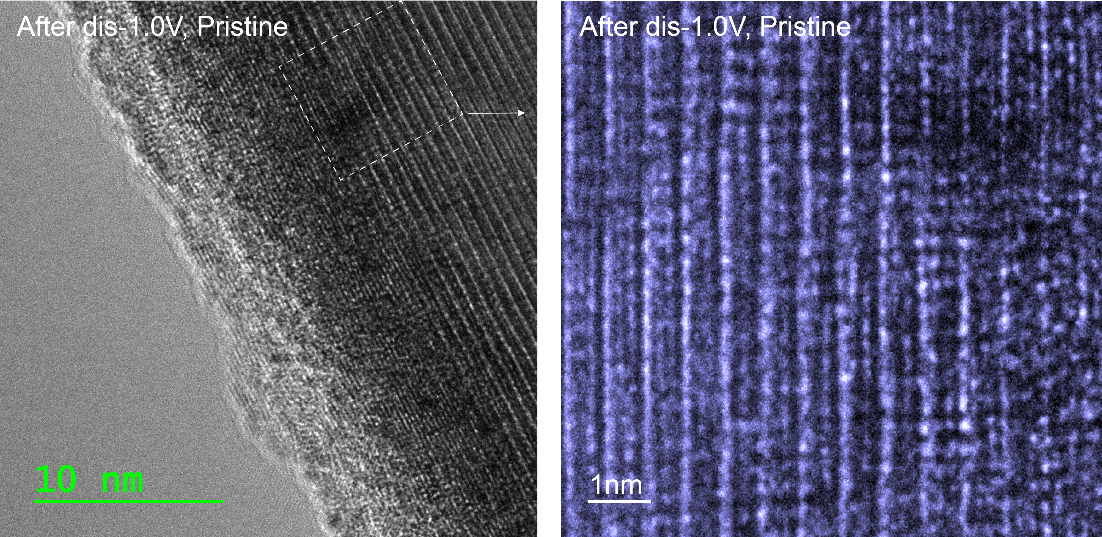


**Figure S8.** HRTEM image of the NVO-based electrode after discharge to 1.0 V in the pristine electrolyte.
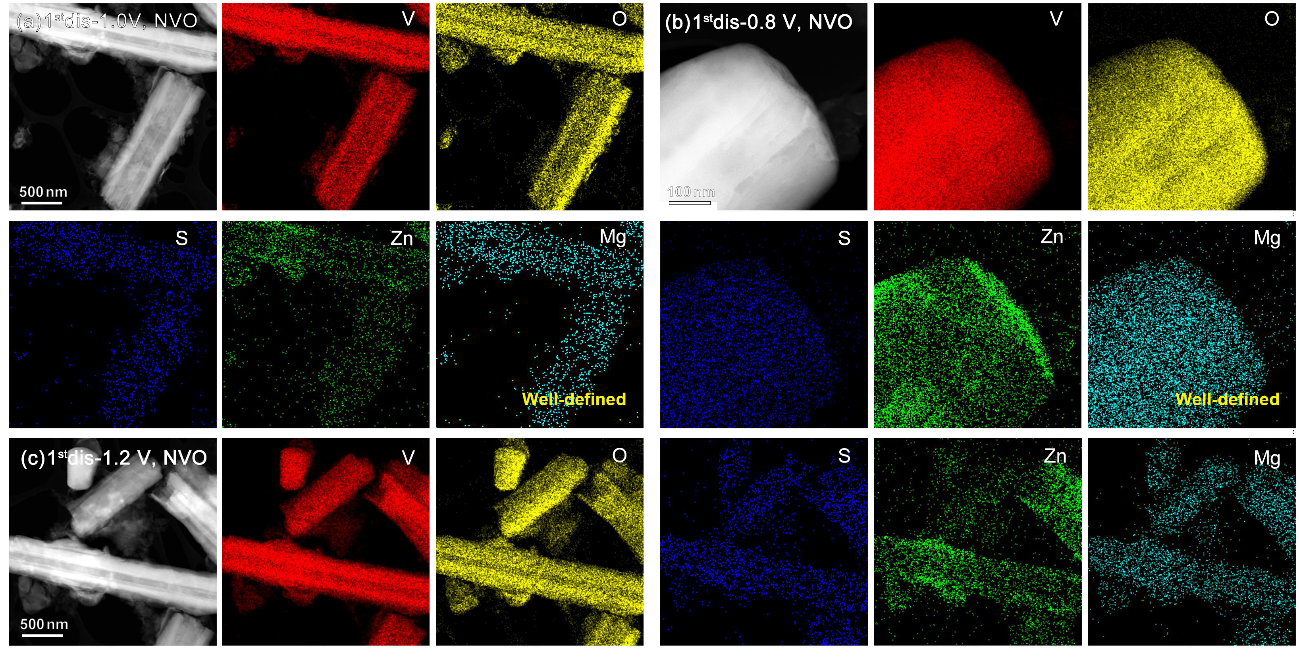


**Figure S9.** HRTEM images and corresponding EDS maps of the NVO electrodes cycled in Mg^2+^-containing electrolytes after the first discharge to 1.0 V (a), 0.8 V (b), and 1.2 (c), respectively.


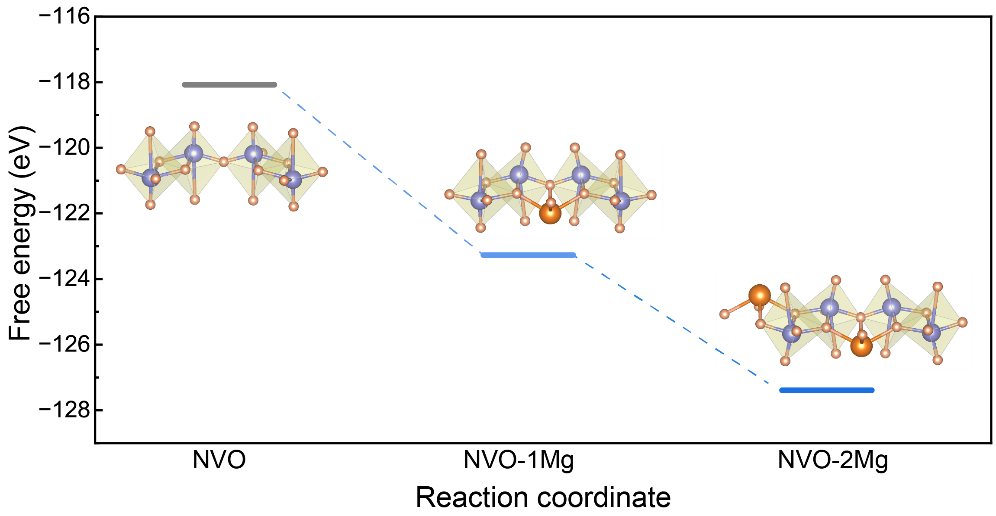


**Figure S10.** Gibbs free energy of Mg^2+^ insertion into the NVO-based cathode.

**Figure S11.** Normalized V K-edge XANES spectra of NVO-based electrodes for the first discharge process.


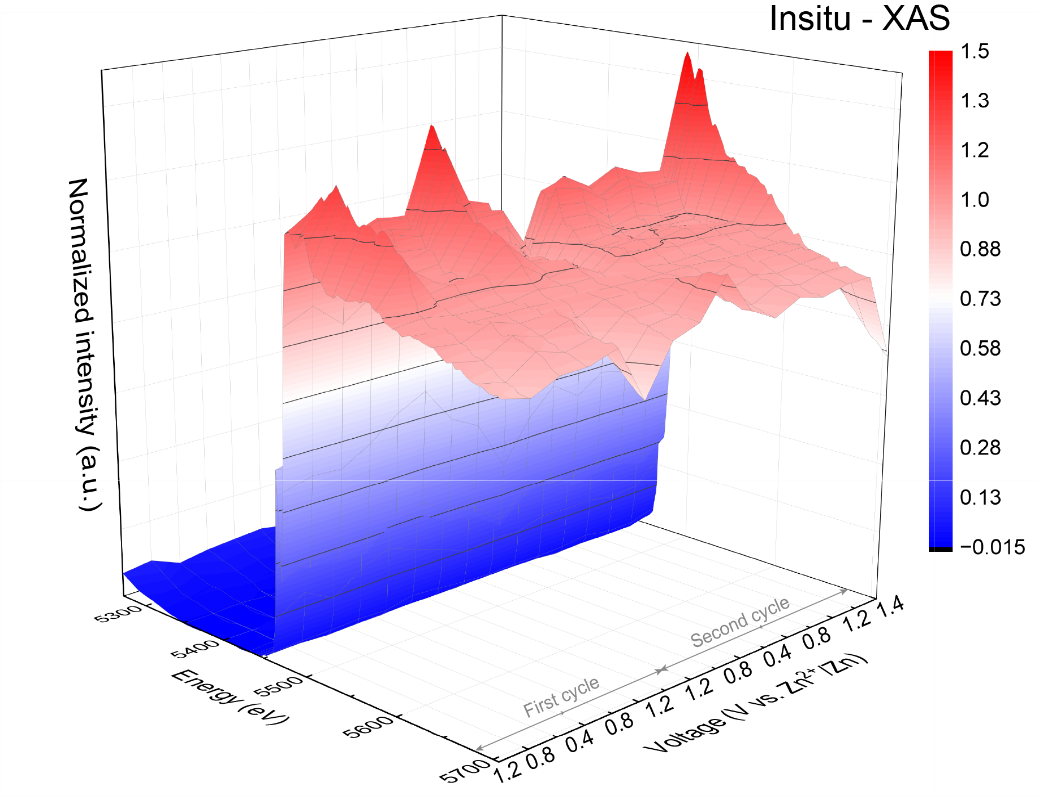


**Figure S12.** In-situ XANES spectra recorded during the first two galvanostatic cycles in tape-type Zn/NVO batteries using the pristine electrolyte.


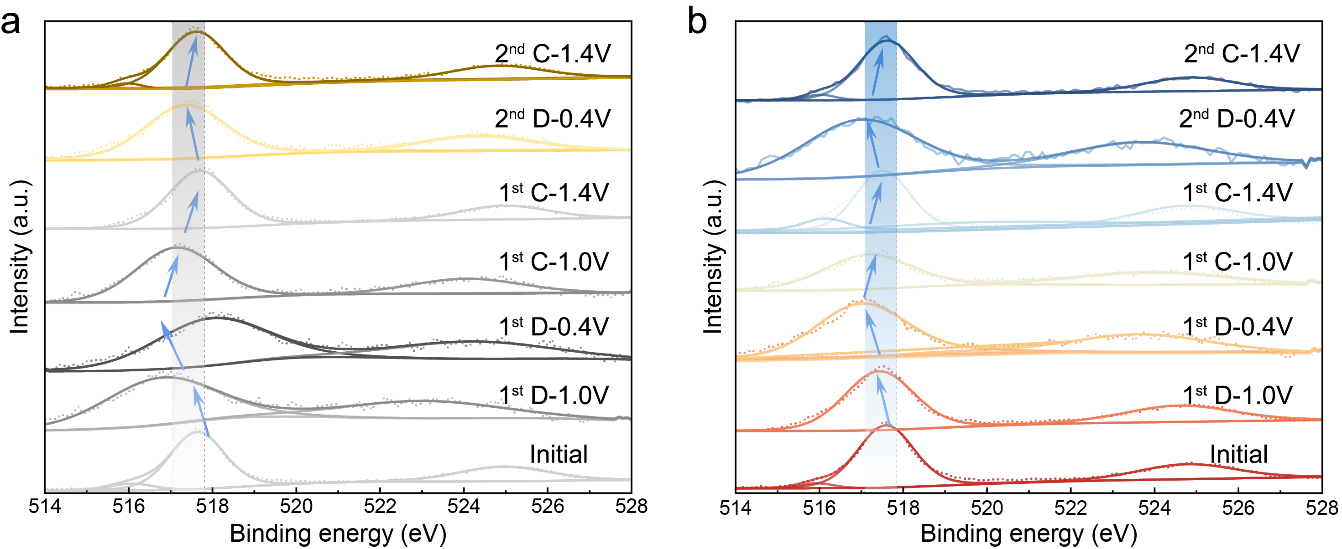


**Figure S13.** XPS analysis of NVO-based electrodes cycled in the pristine electrolytes (a), and Mg^2+^-containing electrolytes (b).


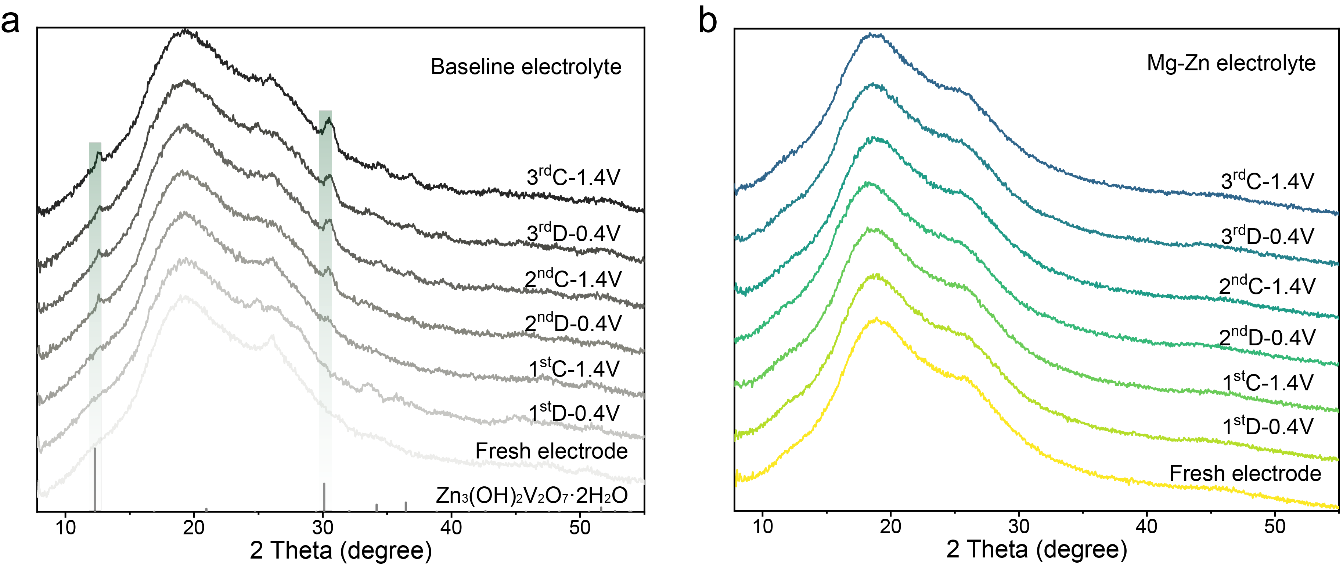


**Figure S14.** XRD patterns selected from the in-situ XRD performance at different cut-off voltages for baseline (a), and the Mg-Zn-based electrolytes (b).


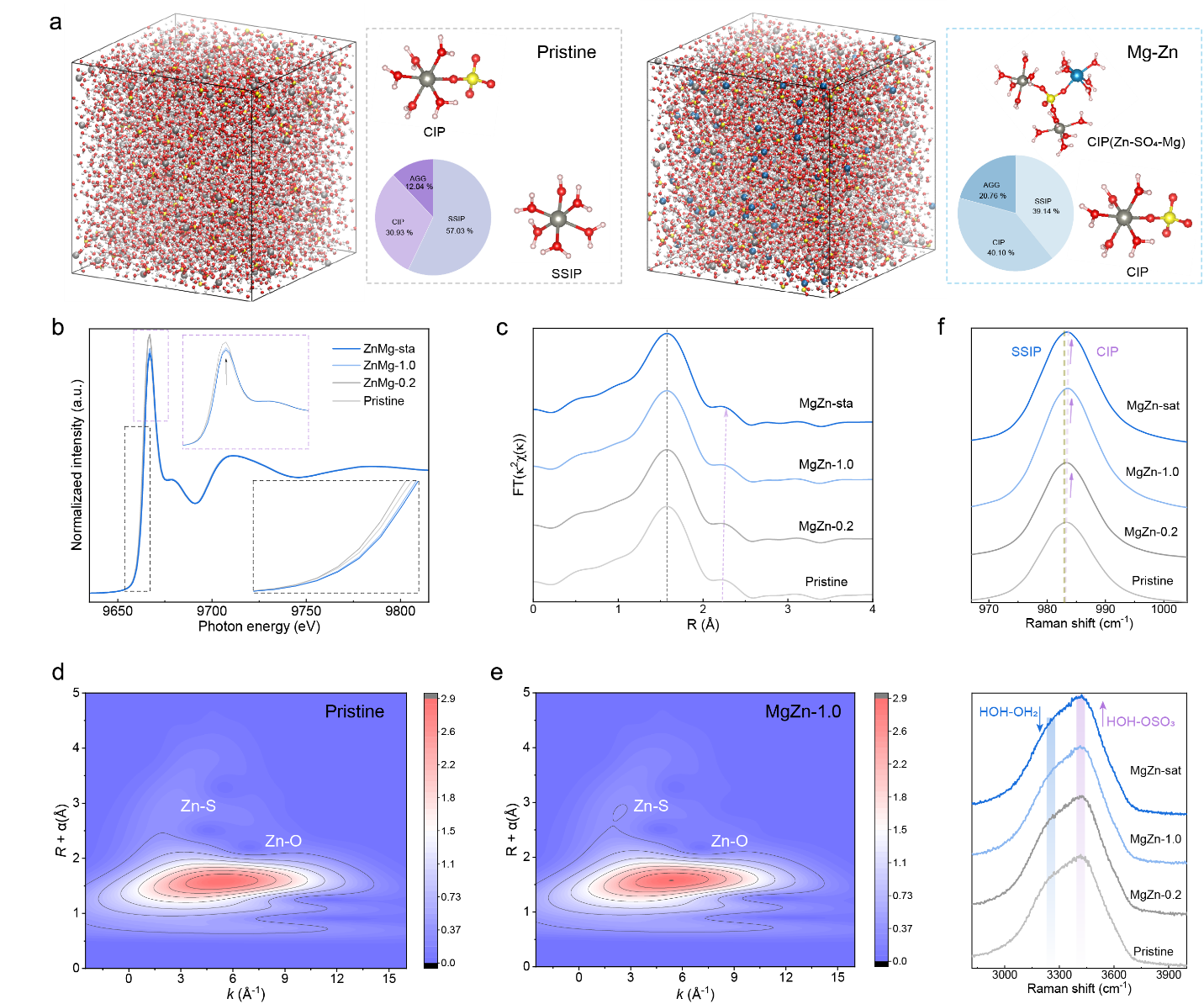


**Figure S15.** (a) 3D snapshot of ZnSO_4_ from MD simulation and corresponding partial enlarged solvated structures and their ion-association ratios for the pristine 2M ZnSO_4_, and Mg-Zn electrolytes, respectively. (b) Normalized *K*-edge XANES spectra of Zn element, and (c) corresponding *K^2^*-weight FT-EXAFS spectra in *R*-space for the pristine electrolyte and Mg^2+^-containing electrolytes with varying Mg concentration. (d) Wavelet transform plots of Zn *K*-edge EXAFS map for the pristine electrolytes (d), and Mg-Zn electrolytes (e). (f) Raman spectra for different electrolytes.

**Figure S16.** Radial distribution function (RDF) plots stemmed from MD simulations for the pristine electrolyte (2M ZnSO_4_) and MgZn-1.0 electrolytes.


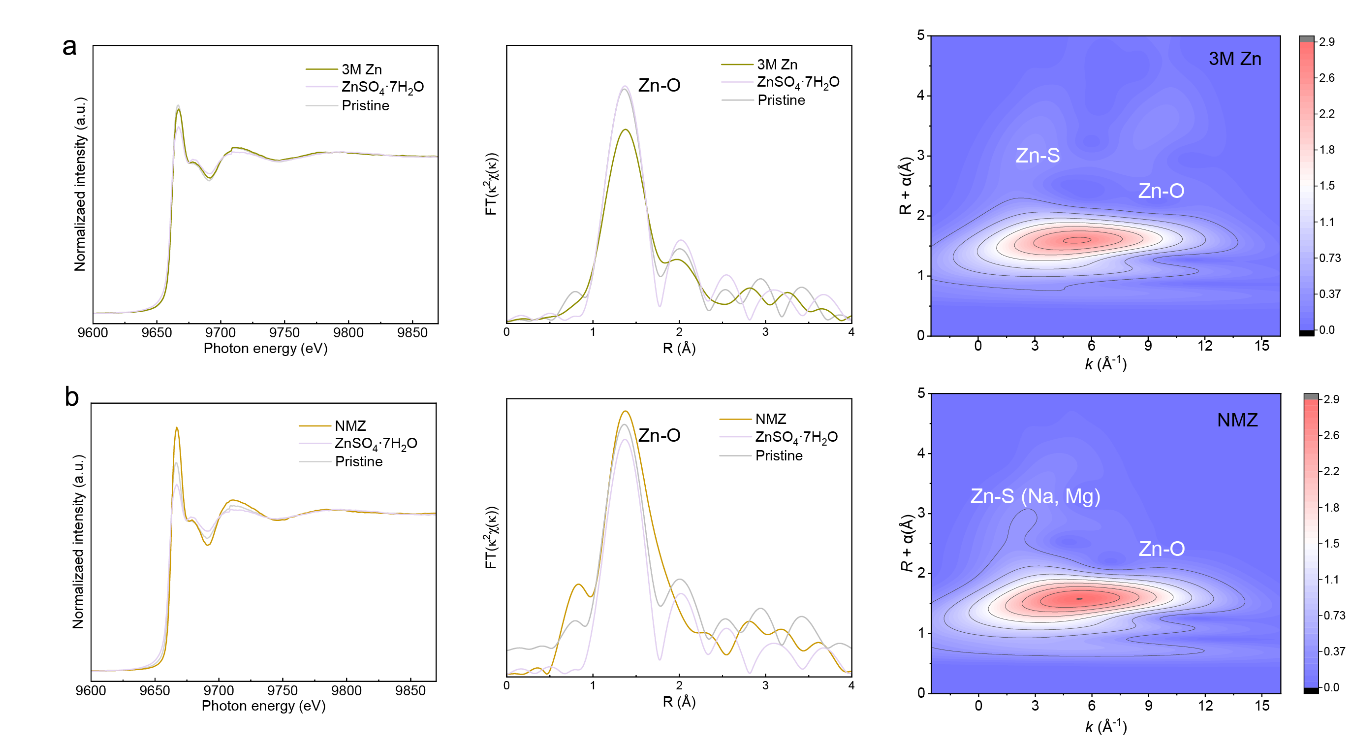


**Figure S17.** Normalized *K*-edge XANES spectra of Zn element, *K^2^*-weight FT-EXAFS spectra in *R*-space, and corresponding wavelet transform plots of Zn K-edge EXAFS data for the 3 M ZnSO_4_ (a), and triple NMZ electrolytes (b).


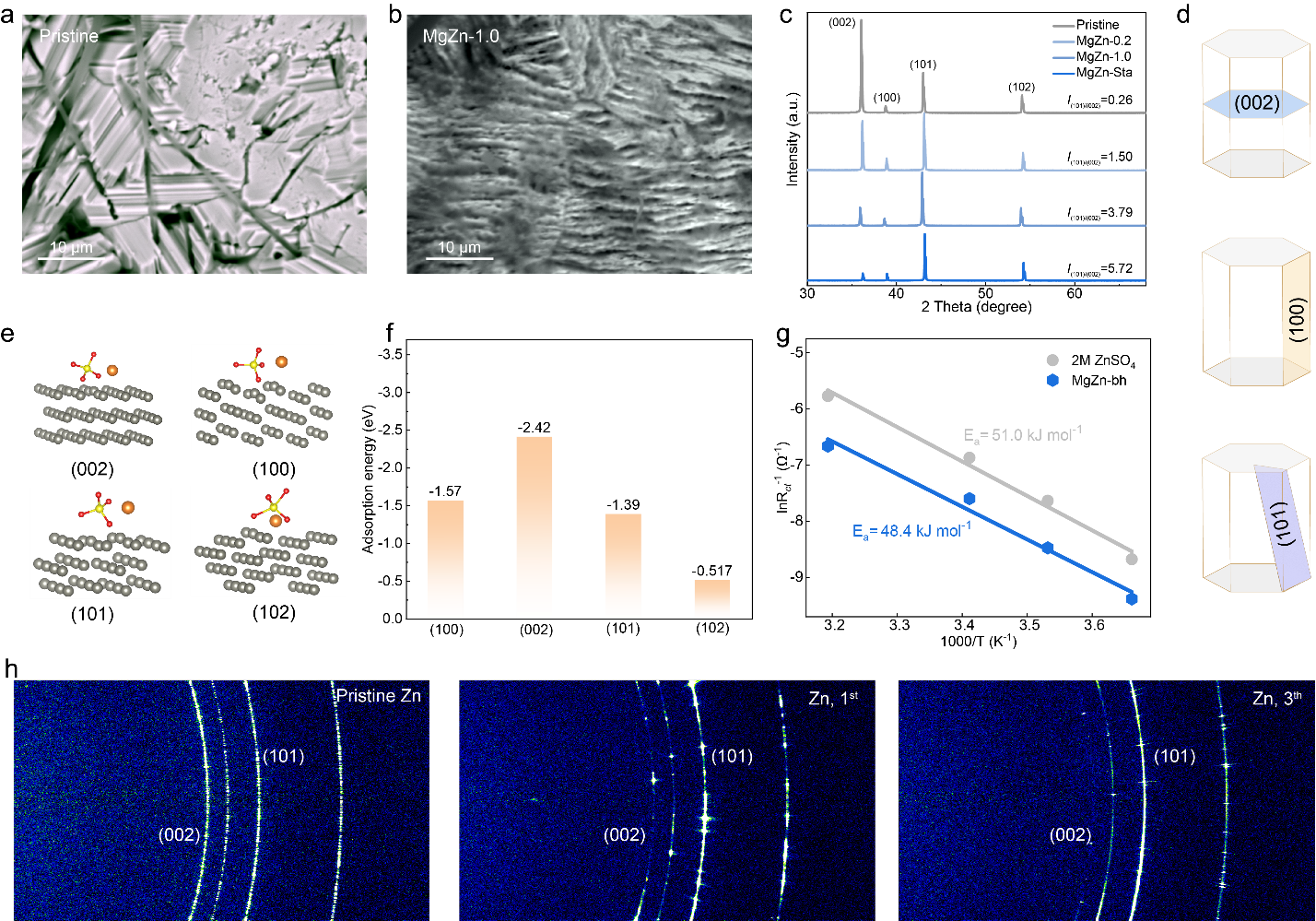


**Figure S18.** SEM images of the zinc anode after three CV cycling in the pristine (a), and MgZn-1.0 electrolytes (b). (c) Corresponding XRD patterns of the anode after cycling. (d) Crystallographic planes within a hexagonal prism of zinc metal anode. (e) Schematic illustration of MgSO_4_ molecules adsorption on different Zn crystalline surfaces, (f) corresponding adsorption energy values. (g) The calculation of activation energies of Zn/Zn symmetric cells with different electrolytes. (h) 2D GIXD patterns of Zn anode cycled with MgZn-1.0 electrolyte after different cycles.


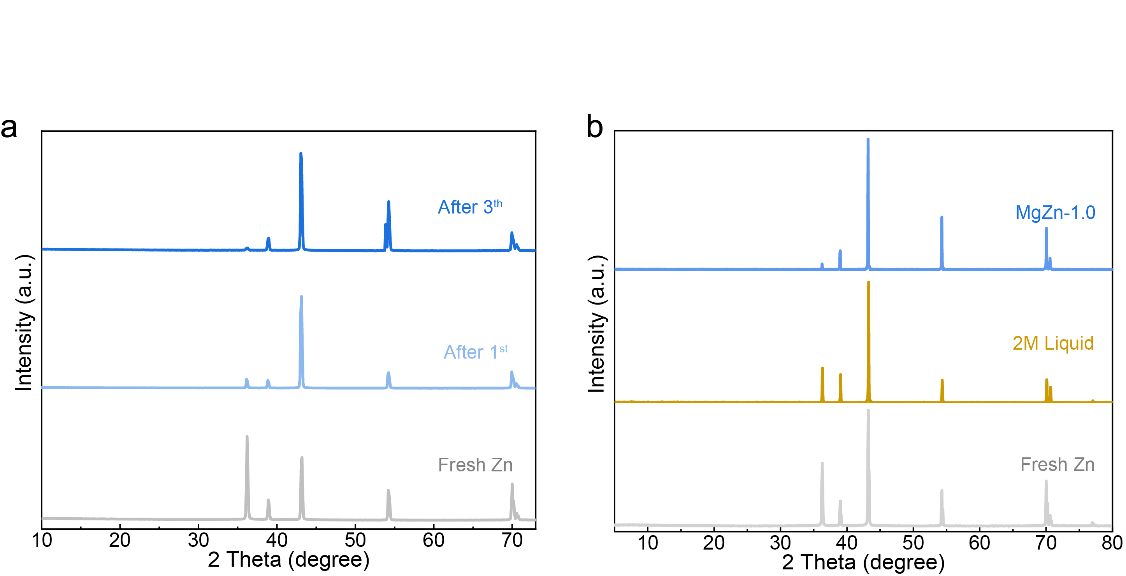


**Figure S19** (a) The integrated XRD patterns stemmed from 2D GIXD results for the Zn anode before and after cycling in the MgZn-1.0 electrolyte. (b) XRD patterns of another commercial Zn anode after three CV cycles at a scant rate of 2 mV s^-1^ for electrode cycle in the pristine and the MgZn-1.0 electrolytes.


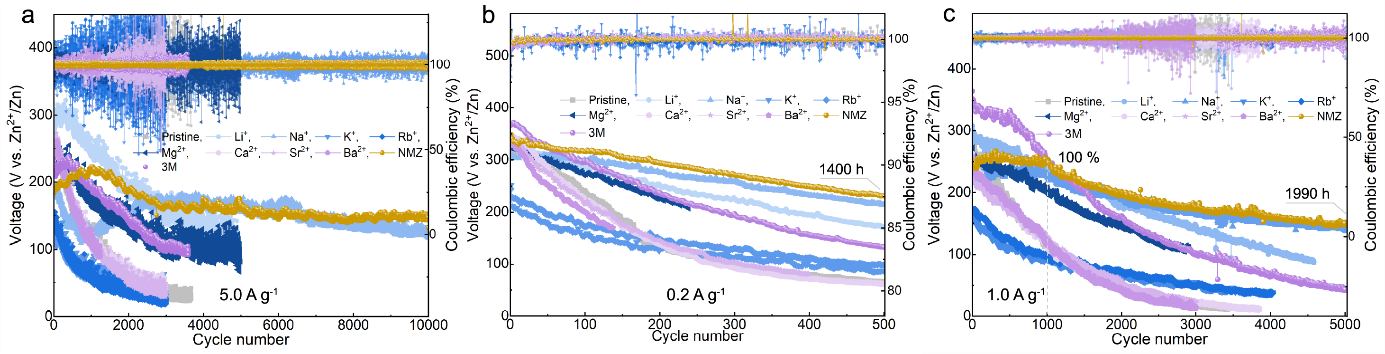


**Figure S20.** Cycling performance of Zn/NVO batteries in the pristine and cation-containing electrolytes at current densities of 5.0 A g^-1^ (a), 0.2 A g^-1^ (b), and 1.0 A g^-1^ (c).


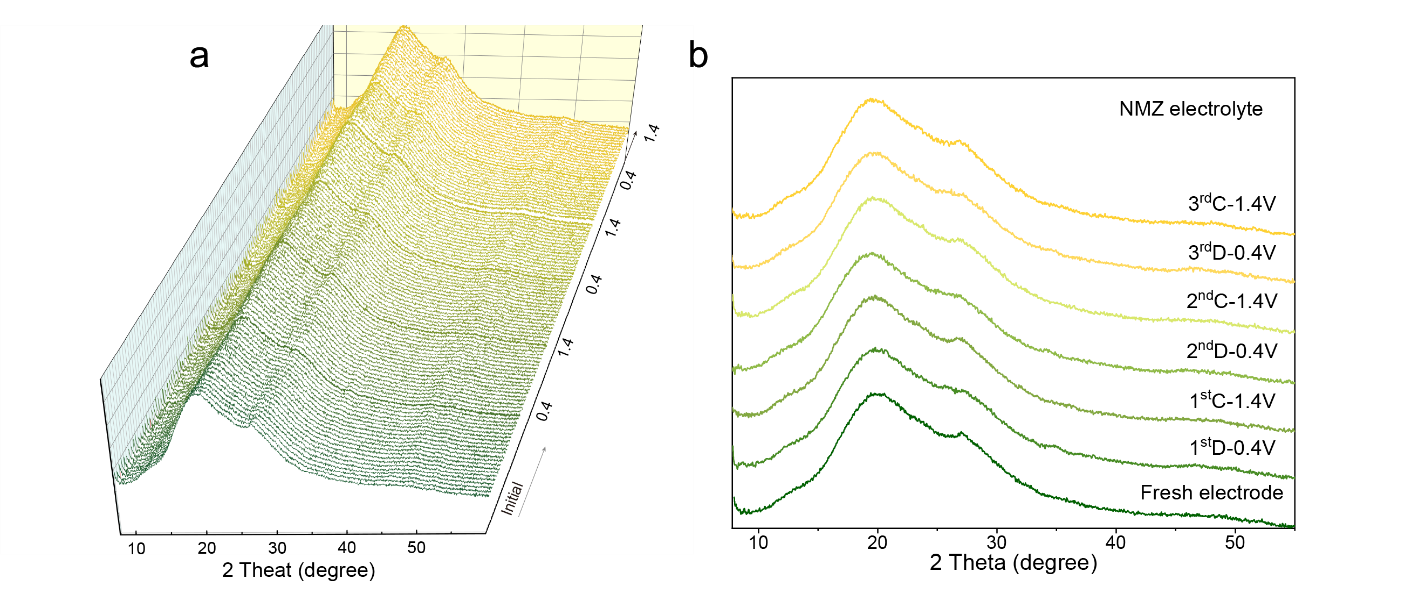


**Figure S21.** In-situ XRD performance of electrode cycled in NMZ electrolyte (a) and corresponding selected XRD patterns at different cut-off voltages.


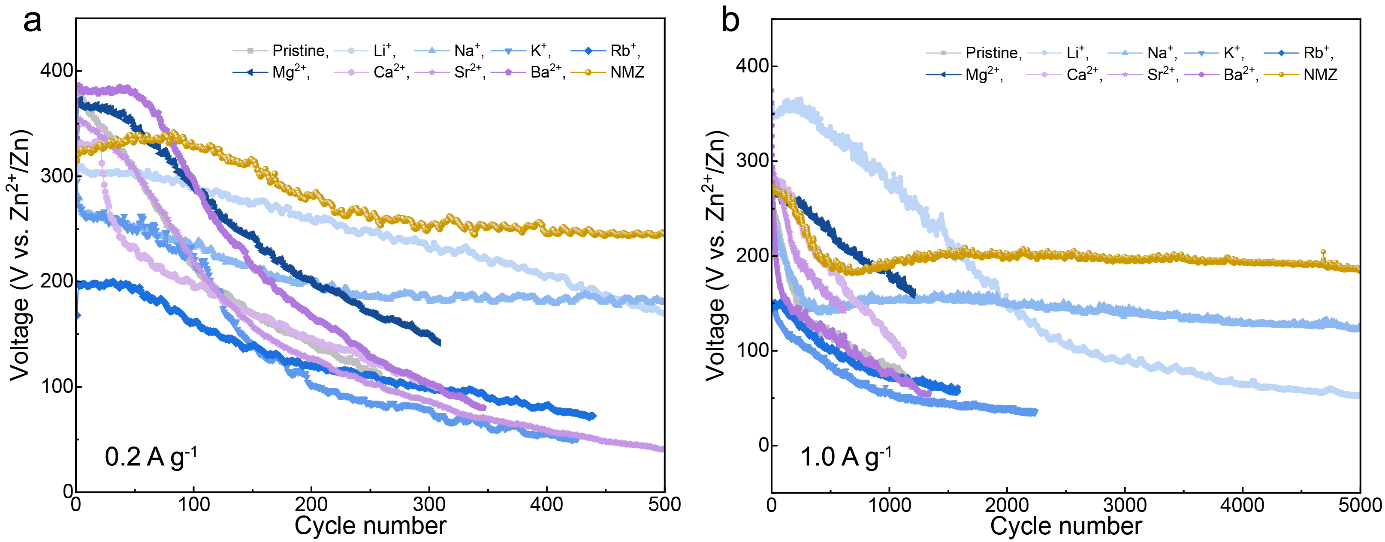


**Figure S22.** Cycling performance of Zn/VO batteries in the pristine and cation-containing electrolytes at current densities of 0.2 A g^-1^(a), and 1.0 A g^-1^(b).


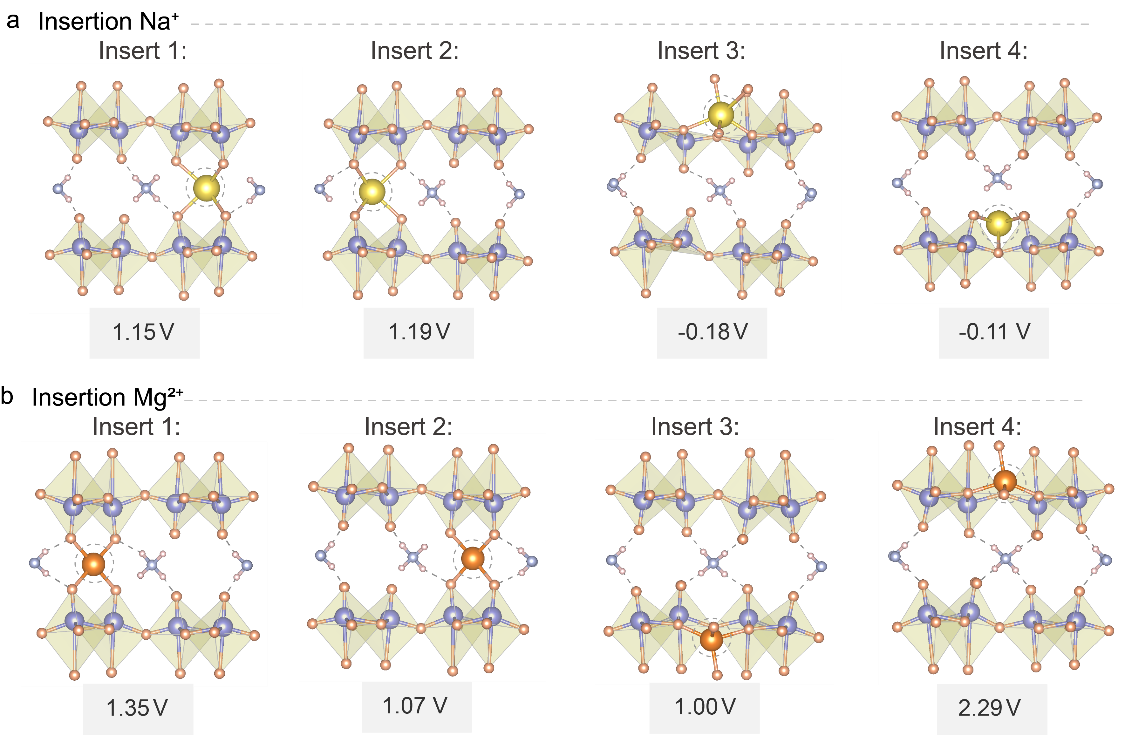


**Figure S23.** Theoretical insertion sites and voltages for Na^+^ (a), and Mg^2+^ (b) inserted into NVO-based materials.

**Table S1.** Comparison of electrochemical performance of vanadium-based cathodes in aqueous electrolytes.

| **Cathode** | **Electrolytes** | **Current density**  **(A cm^-2^)** | **Cycle** | **Capacity retention (%)** | **Reference** |
| --- | --- | --- | --- | --- | --- |
| Na_5_V_12_O_32_ | 1M ZnSO_4_+1M Na_2_SO_4_ | 0.5 | 100 | 66.16 | [4] |
| HNaV_6_O_16_ | 1M ZnSO_4_+1M Na_2_SO_4_ | 0.5 | 100 | 65.13 | [4] |
| V_2_O_5_ | 1M Zn(CF_3_SO_3_)_2_ | 0.5 | 240 | 58 | [5] |
| K_2_V_6_O_16_ | 2M ZnSO_4_ | 0.5 | 50 | 40.23 | [6] |
| Ba_x_V_2_O_5_·H_2_O | 2M ZnSO_4_ | 0.5 | 200 | 33.44 | [7] |
| V_2_O_5_ | 2M ZnSO_4_ | 0.5 | 100 | 26.87 | [8] |
| NaV_3_O_8_·1.5H_2_O | 1M ZnSO_4_ | 0.5 | 300 | 10.6 | [9] |
| Ag_0.4_V_2_O_5_ | 3M ZnSO_4_ | 0.5 | 50 | 72.9 | [10] |
| Na_2_V_6_O_16_ | 3M Zn(CF_3_SO_3_)_2_ | 0.18 | 30 | 89 | [11] |
| V_2_O_5_ | 1M Zn(CF_3_SO_3_)_2_ | 0.2 | 100 | 70.5 | [12] |
| Na_0.33_V_2_O_5_ | 3M Zn(CH_3_F_3_SO_3_)_2_ | 0.2 | 100 | 68 | [13] |
| H_11_Al_2_V_6_O_23.2_ | 2M Zn(ClO_4_)_2_ | 0.2 | 100 | 50 | [14] |
| Li*_x_*V_2_O_5_·nH_2_O | 2M ZnSO_4_ | 1 | 50 | 68.45 |  |
| VOPO_4_·2H_2_O | 2M ZnSO_4_  +NMS | 1 | 500 | 82% | [15] |

**Table S2**. The performance and feature of electrolyte additives based on radar chart cross five key criteria for ZIBs.

| **Electrolytes** | **Conductivity**  **R.T (mS cm^-1^)** | **Stability**  **(1 A g^-1^)** | **Green rate** | **Cost ($/Kg)** | **Safety** | **Reference** |
| --- | --- | --- | --- | --- | --- | --- |
| Pristine | 18.9 | 1000 | 5 | 180 | Good | This work |
| TMS | 26.0 | 600 | 2 | 290 | Bad | [16] |
| TMP | 43.29 | 500 | 2.5 | 246 | Worst | [17] |
| NMP5 | ⁓16 | 2000 | 2 | ⁓200 | Worst | [18] |
| NMZ | 26.0 | 5000 | 5 | 195 | Good | This work |

**References:**

[1] a)G. Kresse, J. Furthmüller, *Comput. Mater. Sci.* **1996**, 6, 15; b)G. Kresse, J. Furthmüller, *Phys. Rev. B* **1996**, 54, 11169.

[2] J. P. Perdew, K. Burke, M. Ernzerhof, *Phys. Rev. Lett.* **1996**, 77, 3865.

[3] G. Kresse, D. Joubert, *Phys. Rev. B* **1999**, 59, 1758.

[4] X. Guo, G. Fang, W. Zhang, J. Zhou, L. Shan, L. Wang, C. Wang, T. Lin, Y. Tang, S. Liang, *Adv. Energy Mater.* **2018**, 8, 1801819.

[5] P. Hu, M. Y. Yan, T. Zhu, X. Wang, X. Wei, J. Li, L. Zhou, Z. Li, L. Chen, L. Mai, *ACS Appl. Mater. Inter.* **2017**, 9, 42717.

[6] B. Y. Tang, G. Z. Fang, J. Zhou, L. Wang, Y. Lei, C. Wang, T. Lin, Y. Tang, S. Liang, *Nano Energy* **2018**, 51, 579.

[7] X. Wang, B. J. Xi, X. J. Ma, Z. Feng, Y. Jia, J. Feng, Y. Qian, S. Xiong, *Nano Lett.* **2020**, 20, 2899.

[8] J. Zhou, L. T. Shan, Z. X. Wu, X. Guo, G. Fang, S. Liang, *Chem. Commun.* **2018**, 54, 4457.

[9] F. Wan, L. L. Zhang, X. Dai, X. Wang, Z. Niu, J. Chen, *Nat. Commun.* **2018**, 9, 1.

[10] L. Shan, Y. Yang, W. Zhang, H. Chen, G. Fang, J. Zhou, S. Liang, *Energy Storage Mater.* **2019**, 18, 10.

[11] P. Hu, T. Zhu, X. Wang, X. Wei, M. Yan, J. Li, W. Luo, W. Yang, W. Zhang, L. Zhou, Z. Zhou, L. Mai, *Nano Lett* **2018**, 18, 1758.

[12] N. Zhang, Y. Dong, M. Jia, X. Bian, Y. Wang, M. Qiu, J. Xu, Y. Liu, L. Jiao, F. Cheng, *ACS Energy Lett.* **2018**, 3, 1366.

[13] P. He, G. Zhang, X. Liao, M. Yan, X. Xu, Q. An, J. Liu, L. Mai, *Adv. Energy Mater.* **2018**, 8, 1702463.

[14] G. Yang, Q. Li, K. Ma, C. Hong, C. Wang, *J. Mater. Chem. A* **2020**, 8, 8084.

[15] Q. Zong, R. Li, J. Wang, Q. Zhang, A. Pan, *Angew. Chem. Int. Ed.* **2024**, 63, e202409957.

[16] Y. Zhong, X. Xie, Z. Zeng, B. Lu, G. Chen, J. Zhou, *Angew. Chem. Int. Ed.* **2023**, 62, e202310577.

[17] W. Wang, S. Chen, X. Liao, R. Huang, F. Wang, J. Chen, Y. Wang, F. Wang, H. Wang, *Nature Commun.* **2023**, 14, 5443.

[18] T. C. Li, Y. Lim, X. L. Li, S. Luo, C. Lin, D. Fang, S. Xia, Y. Wang, H. Y. Yang, *Adv. Energy Mater.* **2022**, 12, 2103231.
